# Future temperature-related mortality in various climate change and adaptation scenarios in Finland

**DOI:** 10.1007/s00484-025-03105-0

**Published:** 2026-03-02

**Authors:** Lisa Haga, Reija Ruuhela, Stefan Fronzek, Nina Pirttioja, Kaisa Lakkala, Timothy R. Carter

**Affiliations:** 1 Meteorological and Marine Research Programme, Weather and Climate Research Impact, Finnish Meteorological Institute (FMI), Helsinki, Finland; 2 Climate Solutions Unit, Finnish Environment Institute (Syke), Helsinki, Finland; 3 Climate Research Programme, Atmospheric Research Centre of Eastern Finland, Finnish Meteorological Institute, Kuopio, Finland; 4 Space and Earth Observation Centre, Earth Observation Research, Sodankylä, Finnish Meteorological Institute, Sodankylä, Finland

**Keywords:** Climate change, Temperature-related mortality, Heat, Distributed lag non-linear model, Adaptation

## Abstract

Human mortality rates are known to be related to ambient temperature and are expected to be affected by climate change. We applied a distributed lag non-linear model (DLNM) to determine regional temperature-mortality relationships in 19 out of 23 Finnish wellbeing service counties, based on daily death and temperature data from the period 2000–2017. We estimated future heat- and cold-related mortality during this century using climate projections assuming moderate (SSP2-4.5) and very high (SSP5-8.5) greenhouse gas emissions. For Helsinki, we also investigated how population projections and adaptation measures might affect temperature-related mortality rates. Our results project a decline in deaths attributable to cold and an increase in deaths attributable to heat during this century over most counties, though with some regional heterogeneity. In 12 out of 19 counties consistent increasing trends in future heat-related mortality were demonstrated with the highest increases in counties in southern and eastern Finland. Also, in 14 out of 19 counties there is consistent decrease in cold-related deaths. Future projections that also account for potential population growth in Helsinki by the end of the century, increase estimates of both heat- and cold-related deaths. However, the results should be interpreted with caution due to substantial uncertainty. There is currently a lack of long-term population projections and associated uncertainties for different regions and age groups in Finland. Further studies on temperature-related mortality should be based on longer time series with a wider range of recent observed temperature extremes as well as more refined sociodemographic predictor variables.

**Supplementary Information:**

The online version contains supplementary material available at 10.1007/s00484-025-03105-0.

## Introduction

An increased heat-related health burden attributable to climate change is already evident in all continents and can be expected to intensify as heatwaves become longer and more frequent in the future (Vicedo-Cabrera et al. 2021; Cissé et al. 2023; Madaniyazi et al. 2024). For example, in one of these studies across 43 countries during the period 1991–2018 (including one location, Helsinki, in the south of the study region here), 37% of warm season heat-related deaths could be attributed to anthropogenic climate change (Vicedo-Cabrera et al. 2021). In Europe during the record-breaking summers of 2023 and 2022, heat caused over 40 000 and 60 000 excess deaths respectively (Lloyd et al. 2023; Gallo et al. 2024). In addition to global warming, future mortality trends depend on many other factors including demographic developments and housing conditions (Cissé and McLeman 2021). For example, in a study from Switzerland it was found that about half of the projected increase in heat-related mortality is caused by climate change and the other half by population developments (de Schrijver et al. 2023a). The group most vulnerable to heatwaves is elderly people with chronic diseases, particularly those living in urban areas. (Ballester et al. 2011; Kollanus & Lanki, 2014, 2021.; Bunker et al. 2016).

Populations are gradually acclimatizing and adapting to climate change and thus the current heat hazard thresholds used in determining risks may not be valid in the future (Huang et al. 2011; Masselot et al. 2025). Various methods for estimating future adaptation exist – for instance, using extrapolation from the historical temperature-mortality trends or adjusting the temperature-related risk function – but to date, no consistent quantitative method has been established for modelling effects of future adaptation on health (Cordiner et al. 2024). On a national scale, Åström et al. 2017 estimated that in Finland a 49.5% reduction in vulnerability across the entire population would be required to maintain heat-related deaths at 1980–2010 reference levels by the 2050 s under a climate projection assuming moderate radiative forcing due to greenhouse gas concentrations in the atmosphere (RCP4.5). Muthers et al. 2010 investigated Austrian mortality data from 1970 to 2007, focusing on long-term adaptation to heat-related risks. They found that if no adaptation takes place, heat-related mortality in Vienna could increase by 129% between 2040 and the end of this century. Even though the risk of heat is significant in most studies (Yang et al. 2021; de Schrijver et al. 2023b) non-optimal temperature such as extreme cold or heat is estimated to cause 7.7% of all deaths and cold is responsible for the majority of deaths, while 0.42% can be attributed to heat (Gasparrini et al. 2015). However, the proportion of deaths attributable to heat is expected to rise as the climate warms in the future.

In Finland, though not considered a high risk, earlier studies have demonstrated a significant impact of heat on mortality (Keatinge et al. 2000; Näyhä 2007; Ruuhela et al. 2018). The prevalence of heatwaves (defined as three or more consecutive days with daily mean temperature > 20 °C) is expected to increase, with mean heatwave duration in southern Finland estimated to increase from 6.1 days, simulated for the 20th century, to 9.4 days under a moderate forcing scenario (RCP4.5) averaged over the 21 st century (Kim et al. 2018) Ruosteenoja and Jylhä 2023a showed that under a 2 °C global warming level relative to pre-industrial times the duration and intensity of European heatwave days is projected to increase three- to fourfold in northern Europe. In southern Finland, a heatwave that occurred once in 10 years during 1977–1996 would occur on average every other year under 2 °C global warming and very severe (1-in-100 year) heatwaves would increase their annual probability to between 9 and 15% (Ruosteenoja and Jylhä 2023b).

In Finland, the estimated excess number of deaths due to cold is annually 1452 deaths and due to heat 45 deaths based on data from nine cities and this accounts for the attributable fraction by cold 8.94% (6.28;11.53) and by heat 0.28% (0.09;0.44) (Masselot et al. 2023). Similar results have been reported in earlier national studies e.g. by Näyhä 2005. Studies on temperature-related mortality show that the impact of cold stress lasted longer (longer lag) than the impact of heat stress which led to a higher overall risk for cold-related than heat-related mortality (Ruuhela et al. 2018). However, during major heatwaves the heat-related excess deaths can still be substantial. For instance, during the heatwaves in 2003, 2010, 2014 and 2018 the estimated heat-related excess deaths varied from 200 to 400 (Kollanus and Lanki 2014, 2021). The totals depend on heatwave duration and intensity, which vary from year to year and geographically in Finland.

More specifically, a range of socio-economic factors may also affect heat-related health risk, by influencing both the hazardous temperatures themselves, but also people’s exposure and vulnerability to them (Landreau et al. 2021). For example, people residing in urban and metropolitan areas are more affected by heatwaves, in part due to the urban heat island (UHI) effect, which enhances temperatures (and hence heat-mortality risk) in the built environment compared to rural areas. For example, in Helsinki the heat-related mortality during heatwaves can be 2.5-times higher than the mortality in the more rural surrounding area (Ruuhela et al. 2021). Moreover, the buildings in which people reside may themselves contribute to enhanced exposure, as in the case of city apartments with no air-conditioning, for which overheating risks are expected to increase in the future (Farahani et al. 2024). Types of building materials, shading and ventilation can moderate inside temperatures, with the quality of housing often related to social status and affordability. Similarly, the most vulnerable members of the population are frequently those with chronic illnesses, the elderly and socially disadvantaged.

Even though heat-related mortality is significant in Finland, sensitivity to heat stress has decreased over the decades, indicating that the Finnish population has adapted to some extent to heat stress. Above the 99th percentile of the thermal index PET (Physiologically Equivalent temperature) distribution, the all-cause, relative mortality for all ages decreased from 18.3 in 1972–1992 to 8.6% in 1994–2014. Similarly, for elderly (75 years and older) the decrease in relative mortality in the highest PET percentile decreased from 21% to 11.1% between these two 21-year periods (Ruuhela et al. 2017, 2018). Ruuhela et al. 2018 investigated regional differences in temperature-mortality relationships in Finland applying the best linear unbiased prediction method to pool different regions together and, thus, reducing the effect of random variation in the relationships. However, this approach converges the relationships towards the average and uses the same minimum mortality temperature across the country, and consequently the approach may also smooth out the existing differences between the regions.

In this study, we aimed to estimate excess cold- and heat-related mortality regionally in the different wellbeing service counties of Finland, hereafter referred to as “counties”, under climate change projections for two emissions scenarios, SSP2-4.5 and SSP5-8.5. We also investigated adaptation scenarios for heat-related mortality and examined how population projections can influence future estimates of temperature-related excess deaths for Helsinki, the most populous region that has some of the highest current rates of heat-related mortality in Finland.

## Materials and methods

### Data

We obtained data on the daily number of all-cause deaths from 2000 to 2017 for 23 wellbeing service counties in Finland (see Fig. SM3), supplied by the Finnish Institute for Health and Welfare (THL). There is no available death information in the data if the observed cases are less than three individuals due to data protection thresholds. The four counties with the lowest total population were also missing data for more than 50% of the days (Table SM1), which we selected as an upper threshold for inclusion in our analysis, hence excluding the counties of Åland, Kainuu, Central Ostrobothnia and East Uusimaa. Our analysis primarily centres on temperature-mortality associations across all cause and all ages mortality. We obtained daily mean temperature data averaged across each county using the Finnish Meteorological Institute’s regular gridded data (FMI_ClimGrid) at 10 km spatial resolution (Aalto et al. 2016).

**Fig. 1:**
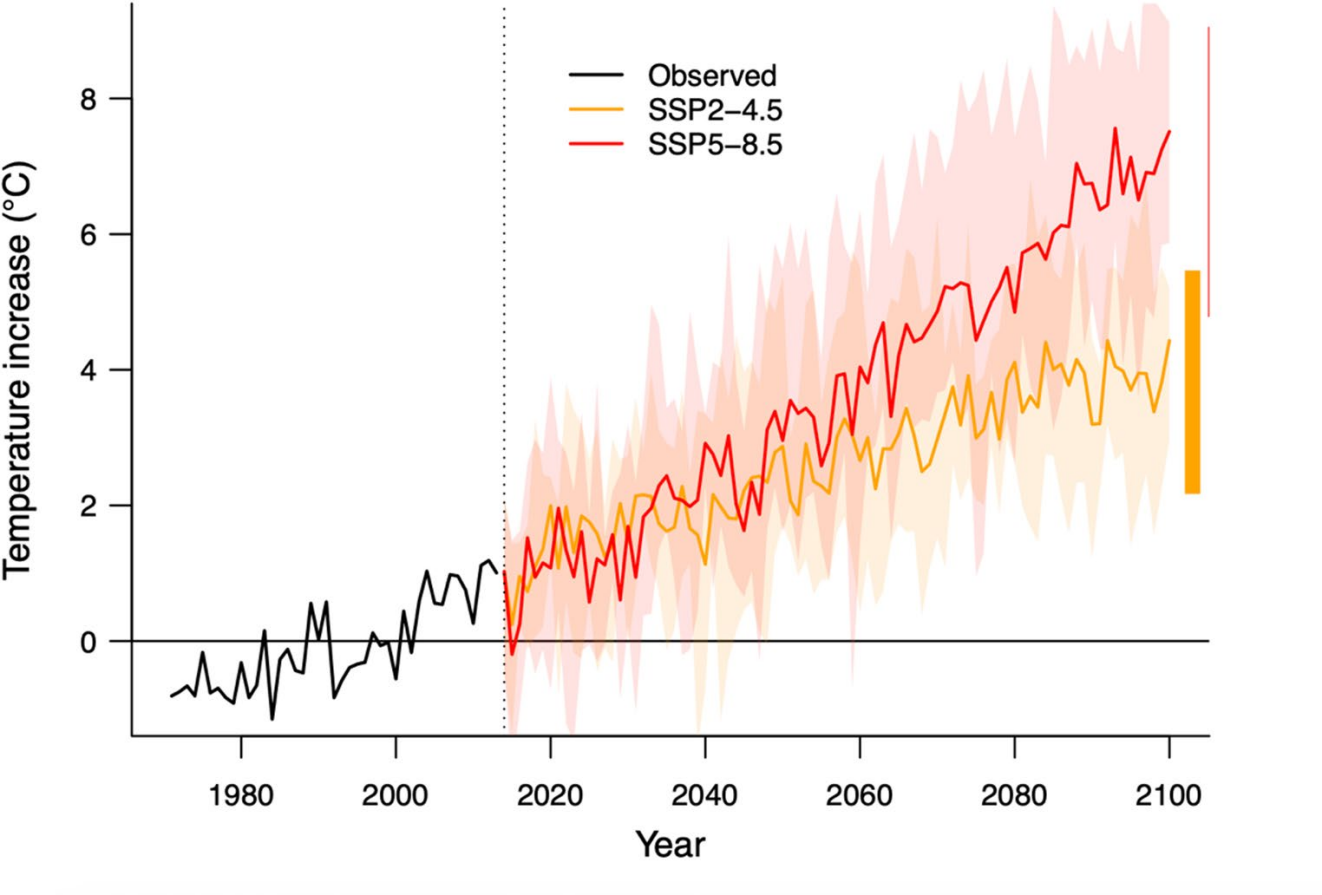
Annual average temperature projections in Finland with CMIP6 climate models under SSP2-4.5 (orange) and SSP5-8.5 (red) scenarios depicted as annual averages (solid lines) for the period 2014–2100 and observed historical values in 1971–2014. The increase in temperature is in comparison to the baseline annual mean temperature on average in 1981–2010. The shaded areas present the climate model variability with maximum and minimum temperatures

Daily future temperature projections for the wellbeing service counties were collected from global climate models (GCMs) participating in Phase 6 of the Coupled Model Intercomparison Project (CMIP6) (Ruosteenoja 2021). We limited the climate projections to results over Finland from five representative, bias-adjusted climate models for two greenhouse gas emission scenarios. A moderate scenario, SSP2-4.5, is estimated to be achievable if emissions reduction commitments made by various countries around the world were to be realized. A high emissions scenario, SSP5-8.5, explores the upper levels of plausible future temperature change projections, which are relevant for informing precautionary decision-making considering the most extreme conditions. Selection criteria for model projections were based on evaluation scores for the representativeness of modelled temperature and precipitation over Finland, with all five models receiving good scores in the evaluation. More information about the climate model selection is found in Sect. 1 of Supplementary material. Figure 1 presents annual mean temperature projections for Finland under SSP2-4.5, and SSP5-8.5 emission scenarios based on the five selected CMIP6 models. Relative to the 30-year baseline period of 1981–2010, across the five models, the projected increase in annual mean temperature by the end of the century is 2.2–5.5 °C under the SSP2-4.5 scenario and 5.8–9.1 °C for SSP5-8.5.

Population projection data were obtained from the International Institute for Applied Systems Analysis (Huppmann et al. 2018. version 2.0: https://tntcat.iiasa.ac.at/SspDb), comprising country-level projections at 5-year intervals under five different Shared Socioeconomic Pathways (SSPs) (Kc and Lutz 2017). In the results we present population projections from SSP2, which represents a middle of the road scenario where the global population growth is moderate. Here, Finland’s population increases from 5.4 million in 2010 to 6.8 million in 2100. We pair projections of SSP2 population with SSP2-4.5 climate. Additionally, we use the fossil-fuel reliant, high emission scenario SSP5, with Finnish population projected to surpass 10 million by 2100, pairing projections of SSP5 population with SSP5-8.5 climate. Though SSP population projections were recently updated by IIASA, our earlier calculations for version 2.0 projections are valid for illustrating future sensitivity of mortality estimates to population.

There is a lack of sub-national population projections for counties until the year 2100. Therefore, we limited our future modelling to projections for Helsinki county alone, assuming that the population in Helsinki would grow at a similar rate as population projections for Finland as a whole. In addition, for computing the total number of future estimated annual deaths, included later in the modelling to calculate excess heat and cold related deaths, we used death rates as a proportion of Helsinki population reported during 2016–2020 (Statistics Finland, https://pxdata.stat.fi/PxWeb/pxweb/fi/StatFin/) as a reference for scaling future values.

### Statistical methods

We estimated temperature-mortality relationships across different counties using a statistical regression modelling approach, the distributed lag nonlinear model (DLNM) (Gasparrini et al.^ 2010^). To estimate projected future excess deaths attributable to heat and cold, we utilized a statistical methodology for time-series analysis in environmental epidemiology developed by Vicedo-Cabrera et al. (2019).

The first step was to define the temperature-mortality relationships for every county relative to the minimum mortality temperature using DLNM with a quasi-Poisson family. The minimum mortality temperature (MMT) values used in the modelling were based on previous studies in Finland, e.g. by Ruuhela et al. (2018). This MMT threshold value varies from 16 °C in southern Finland to 14 °C in the north (see Table SM1, supplementary material). We used a 21-day lag, as cold-related impacts on mortality in Finnish population may last several weeks (Ruuhela et al. 2018). Such a long lag time also accounts for short-term mortality displacement (harvesting effect) due to heat stress. To control long-term trends we used a natural cubic spline with 7 degrees of freedom. We used two different models to estimate the relative risks. The first model used fixed knots with a natural cubic spline as applied in earlier studies (e.g., de Schrijver et al. 2023b). This had three internal knots at the 10th, 75th and 90th percentiles for temperature, and on the lag scale we used a natural cubic spline with two internal knots. The second model used the Quasi-Akaike Information Criteria (qAIC) to estimate the best knot alternative for the exposure variable and lag (see Supplementary Tables SM3-5). In the supplementary material, we present additional sensitivity analysis and relative RMSE results from the counties (see Tables SM2-SM5).

The general formula to estimate the exposure-response relationship over different lags, i.e. the association between daily deaths and temperature, was calculated with the following equation, 1.

1 $$\:log\left[E{Y}_{t}\right]=\alpha\:+f\left({\chi\:}_{t};\theta\:\right)+s\left(t;\beta\:\right)+{\sum\:}_{P=1}^{P}{h}_{p}\left({z}_{pt};{\gamma\:}_{p}\right)$$

Here, $\:{Y}_{t}$ relates to daily death counts presumed to follow a Poisson distribution with overdispersion against time *t*. The term $\:f\left({\chi\:}_{t};\theta\:\right)$presents the exposure-response function between temperature and daily deaths over time *t*, $\:\theta\:$ specifies the parameters for the response function and $\:\alpha\:\:$is the intercept. The term *s(*$\:t;\beta\:$*)* describes the baseline trend that accounts for the long-term and seasonal variation over time *t* and $\:\beta\:$ controls temporal trends. $\:{\sum\:}_{P=1}^{P}{h}_{p}\left({z}_{pt};{\gamma\:}_{p}\right)\:$presents other possible covariates; here the effect of day of the week was included in the analysis.

To determine the sum of excess future deaths for certain time intervals, we calculated the daily number of deaths attributed to heat and cold based on the estimated relative risk and exposures. First, we computed for each day the number of cases attributed to temperature based on the estimated risk at the level of exposure on that day. After this we calculated the future attributable number by using the predicted risk (future scenarios for daily temperature) and the attributable numbers were aggregated to future ten-year time intervals. The uncertainty limits were calculated with Monte Carlo simulations, and we report the 95% empirical confidence intervals (CI) in the results. Lastly, we repeated the analysis by using projections of the number of deaths based on the population projections for Helsinki to assess their effect on temperature-related mortality.

Future projected excess heat and cold attributable number of related deaths was calculated according to Eq. 2:

2 $$\:{D}_{attr}=D\left(1-{e}^{-\left({f}^{*}\left({T}_{mod}^{*};{\theta\:}_{b}^{*}\right)\:-{s}^{*}\left({T}_{mm};{\theta\:}_{b}^{*}\right)\right)}\right)$$

Where $\:{D}_{attr}$ stands for the attributable number of deaths at non-optimal temperature. *D* stands for the total observed deaths, f* for the effect of modified temperature $\:{T}_{mod}^{*}$ from climate change scenarios and s* for the effect of deviation from the minimum mortality temperature, $\:{T}_{mm}$, to quantify the excess risk of non-optimal temperatures. In the results we present aggregated daily number of deaths or fraction of deaths attributable to heat or cold by ten-year intervals until 2090-99 as compared to the time period 2010–2019. The relative attributable fraction (AF%) represents the excess heat or cold-related deaths as a percentage of total deaths in 2090–2099 compared to the equivalent percentage for the time period 2010–2019.

We used two scenarios to estimate plausible adaptation to heat in the future, assuming that adaptation would decrease heat-related relative risk relative to the present by 50% and by 20%, respectively. The justification for the higher rate of adaptation is based on the results of Ruuhela et al. (2017), which show an historical decline of 50% in heat-related mortality risk in Finland since the 1970s. To study a low-adaptation scenario we used a reduction of 20% in heat-related mortality that has been used in some other adaptation studies, such as by Petkova et al. (2017) and Lee et al. (2019).

## Results

### Temperature-mortality relationships

**Fig. 2:**
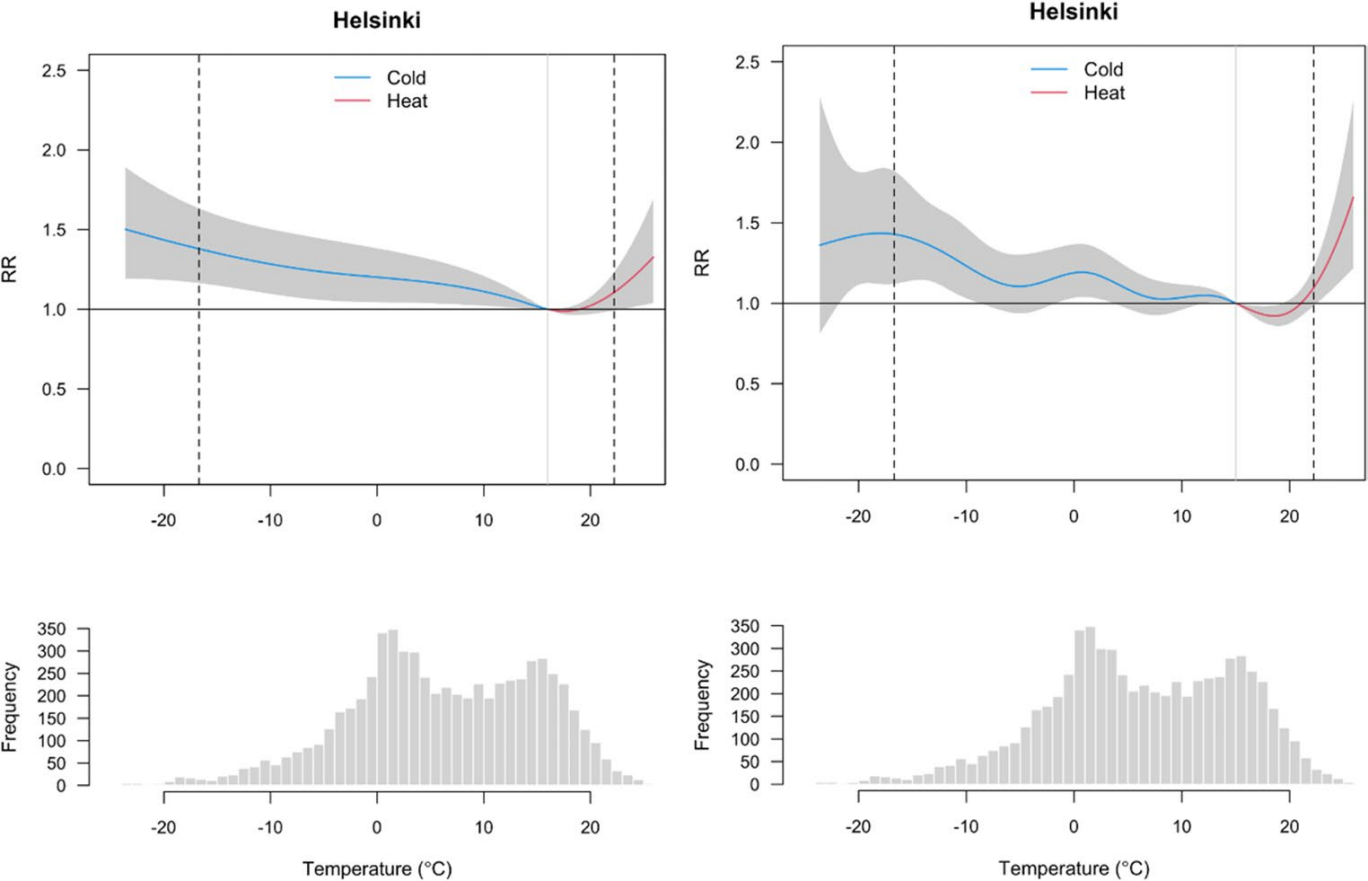
Overall relative risks (RR) of temperature-related mortality and daily mean temperature distributions in Helsinki wellbeing service counties in 2000–2017. In the upper graphs the grey area illustrates the 95%-CI (confidence interval) of the modelled relative risk associated with heat (in red) and cold (in blue). The dashed vertical lines present 1 st and 99th percentiles and MMT is presented as a solid vertical line. The lower graph presents the frequency distribution of daily mean temperature in 2000–2017

In the first phase of the analysis, we studied the associations between observed temperature and daily deaths with two different modelling approaches. Figure 2 illustrates the overall temperature-mortality relationships in the county of Helsinki based on the fixed knots model (left panels) and qAIC knots model (right panels) during the period 2000–2017 and accumulated using a 21-day lag period. The two models produce similar shapes for the temperature-mortality association, but the confidence intervals are wide. However, the model selection has an influence on the relative risk especially on the heat side and, consequently on future estimates as well. Temperature-mortality associations for all counties are shown in Fig. SM1 in the Supplementary material. Our results indicate that the relative risk (RR) typically increases with low and high temperatures compared to the MMT, but with large variations between counties. In some counties (South Savo, South Ostrobothnia and Central Finland) neither of the modelling options showed increase in heat-related risk at the highest temperatures which cannot be considered realistic.

### Future excess heat and cold related mortality projections

**Fig. 3:**
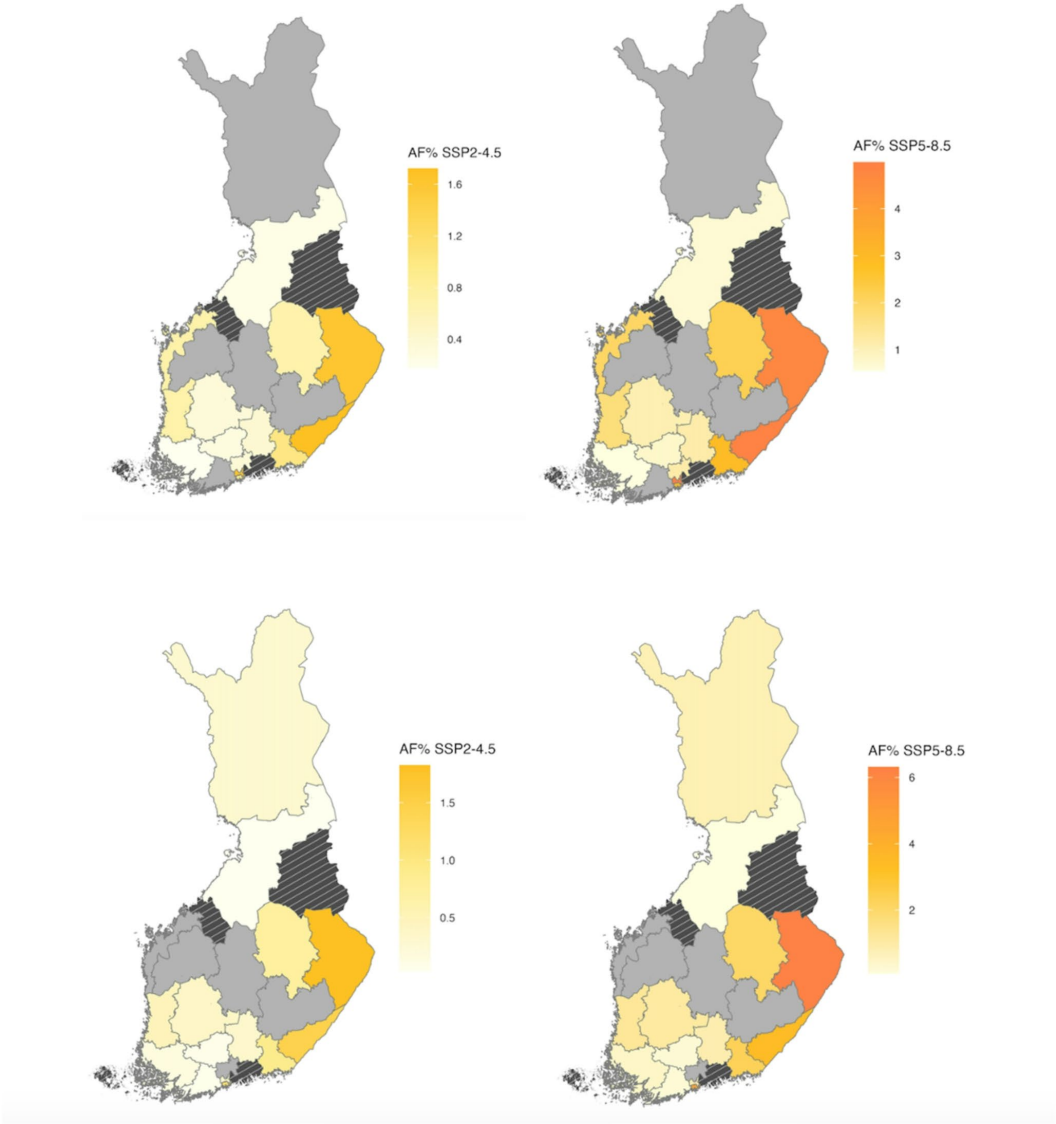
Relative attributable fraction (AF%) of all-age heat-related deaths for Finnish wellbeing service counties under SSP2-4.5 (left) and SSP5-8.5 (right) scenarios in 2090–2099 compared to the period 2010–2019. Estimates are based on fixed knots models (upper panels) and qAIC knots models (lower panels). Yellow to red shades indicate an increase in AF. In grey-coloured counties the model did not show an increase in heat-related deaths. Striped counties were excluded from the analysis

The future changes in deaths attributable to heat and cold were studied as attributable fraction or number of excess deaths. Figure 3 presents the relative attributable fraction (AF%) to heat in all-cause, all-age mortality between 2010 and 2019 and the future period of 2090–2099 under the SSP2-4.5 (left panels) and SSP5-8.5 (right panels) scenarios using the fixed knot model (upper panels) and qAIC knot model (lower panels). The AF% of the heat-related mortality is projected to increase in most of the counties under both SSP2-4.5 and SSP5-8.5. Compared to 2010–2019, the highest increase in heat-attributable mortality is in the counties in southern and eastern Finland, with an approximately 4% increase in excess deaths due to heat under the high emission scenario. The model with fixed knots projected an increase in heat-related deaths in 14 counties and the model with qAIC knots in 15 counties. Model choice influences the results substantially for Lapland, West- and Central Uusimaa and Ostrobothnia. On the other hand, the models provide consistent estimates for future heat-related deaths for Helsinki, North Karelia and Pirkanmaa. The counties of South Savo, South Ostrobothnia and Central Finland show decreasing trends under both model options (grey coloured counties in central Finland in Fig. 3).

**Fig. 4:**
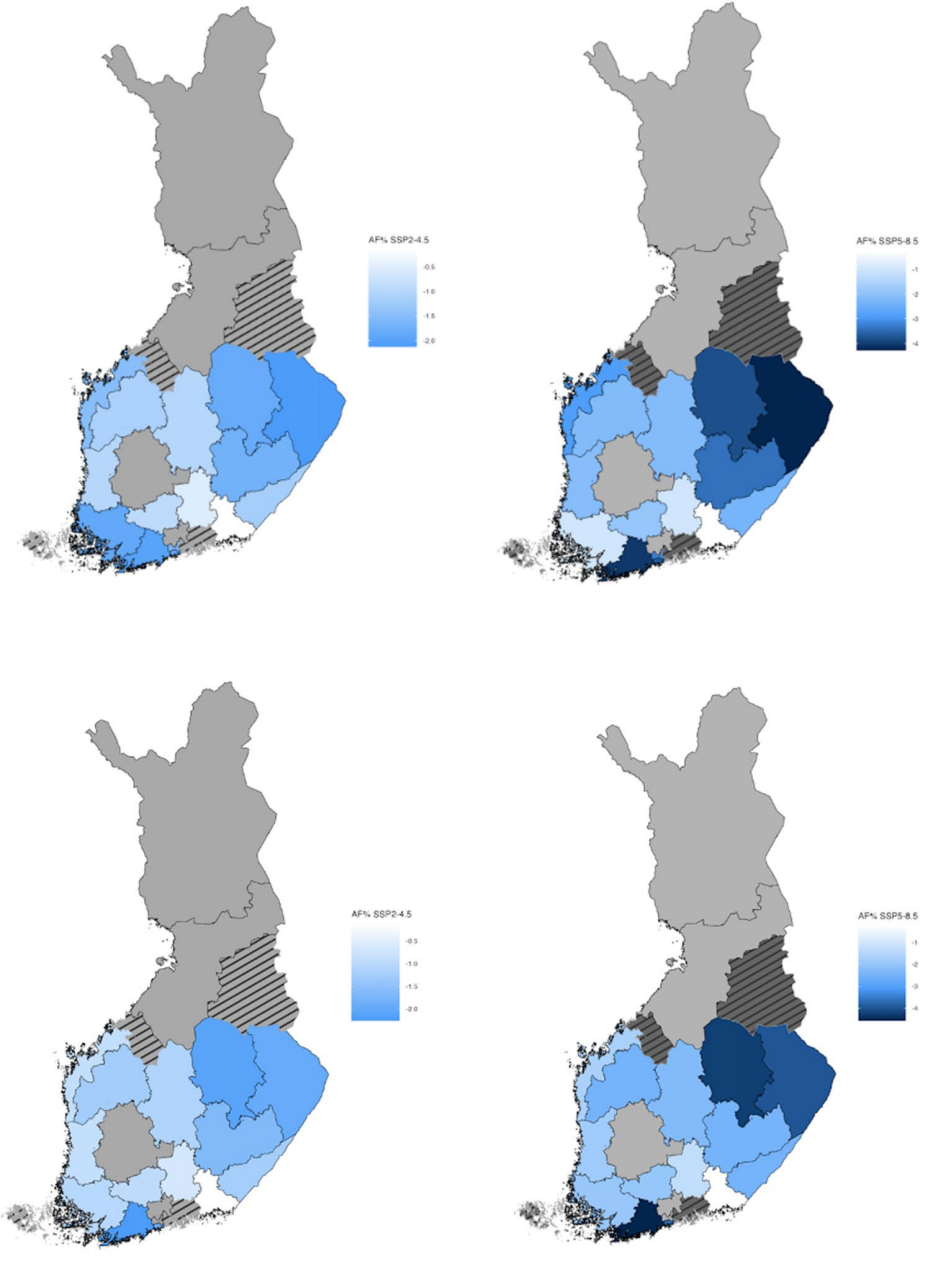
Relative attributable fraction (AF%) in all-age cold-related deaths under the SSP2-4.5 (left) and SSP5-8.5 (right) scenarios by 2090–2099 compared to 2010–2019. Blue shades indicate a decrease in AF. Upper panels show results for fixed knots models and lower panels for qAIC models. In grey-coloured counties the model did not show decreases in cold-related deaths. Striped counties were excluded from the analysis

Similarly, Fig. 4 illustrates county-level projections of cold-related AF% under SSP2-4.5 and SSP5-8.5 scenarios. Projected all-age cold-related AF decreases by 2090-99 compared to 2010–2019 in most of the counties except in Pirkanmaa, Central Uusimaa, Vantaa and Kerava, Northern Ostrobothnia and Lapland. For both models, in 14 out of 19 counties the cold-related AF% is expected to decrease. The largest decrease in cold-related deaths, more than 4% as attributable fraction, is projected in eastern Finland and some southern coastal counties.

As excess deaths due to heat are expected to increase, we also calculated the increase as numbers of deaths attributable to heat to better understand the future societal challenges of heat-related health risks. Table 1 shows the numbers of deaths attributable to heat (AN) for selected ten-year time periods for all counties under SSP2-4.5 and SSP5-8.5 scenarios until the end of the century for the fixed knots model. The selected time periods are 2010–2019, 2050–2059 and 2090–2099. In addition, we calculated the magnitude of the projected change as a ratio for the end of the century relative to the baseline value in the different regions and scenarios (Table 1, last columns). Based on the results in Table 1, and comparable results for the qAIC knots model in the Supplementary Material (Table SM6-8), we conclude that the magnitude of change illustrates different results between the model options, and these can only indicate an increase or decrease in the future heat-related deaths.

**Table 1:** All-age excess heat-related attributable number of deaths (AN) for wellbeing service counties in Finland with 95% confidence interval in parentheses. The results include fixed knots model projections for 2010–2019, 2050–2059 and 2090–2099 under both SSP2-4.5 and SSP5-8.5 emissions scenario. Ratios are for end of the century relative to baseline AN values

	AN Heat under SSP2-4.5				AN heat under SSP5-8.5				
Wellbeing service county	1 2010–2019 SSP2-4.5	2 2050–2059 SSP2-4.5	3 2090–2099 SSP2-4.5	Ratio of columns 3:1	4 2010–2019 SSP5-8.5		5 2050–2059 SSP5-8.5	6 2090–2099 SSP5-8.5	Ratio of columns 6:4
Central Uusimaa	22 (−102;135)	48 (−192;292)	76 (−301;470)	3.4	25 (−113;153)		70 (−261;411)	201 (−704;1033)	8.0
West Uusimaa	−21 (−235;165)	−64 (−620;320)	−113 (−1029;494)	-	−26 (−277;180)		−102 (−873;421)	−350 (−2714;1049)	-
Vantaa & Kerava	119 (−7;246)	245 (9;570)	378 (21;887)	3.2	136 (−3;283)		343 (28;787)	905 (137;1891)	6.6
Helsinki	**107** **(−164; 368)**	**315** **(−167;969)**	**540** **(−181;1647)**	**5.0**	**136** **(−156;449)**		**497** **(−150;1434)**	**1577** **(−85;3890)**	**11.6**
Kymenlaakso	107 (−64;265)	204 (−99;562)	313 (−142;897)	2.9	117 (−66;294)		279 (−128;790)	718 (−341;1850)	6.1
Päijät-Häme	47 (−163;239)	88 (−285;443)	131 (−439;698)	2.8	52 (−175;258)		120 (−395;611)	300 (−1059;1437)	5.7
South Karelia	172 (−0.7;341)	306 (15;647)	457 (33;1010)	2.7	184 (1;363)		408 (34;898)	993 (130;1979)	5.4
Pirkanmaa	54 (−240;323)	125 (−435;702)	202 (−665;1124)	3.7	62 (−263;366)		186 (−585;996)	532 (−1511;2465)	8.5
South Ostrobothnia	−120 (−348;50)	−194 (−717;139)	−277 (−1178;255)	-	−128 (−389;61)		−243 (−1014;218)	−501 (−2541;716)	-
Kanta-Häme	30 (−152;199)	54 (−295;383)	79 (−476;588)	2.6	32 (−174;222)		72 (−420;520)	174 (−1183;1260)	5.4
Satakunta	130 (−54;323)	214 (−149;638)	301 (−269;990)	2.3	139 (−64;37)		266 (−233;870)	562 (−837;1941)	4.0
Southwest Finland	28 (−228;267)	67 (−475;594)	107 (−754;979)	3.8	33 (−253;306)		96 (−660;825)	276 (−1839;2146)	8.4
Ostrobothnia	34 (−107;173)	91 (−204;420)	156 (−315;706)	4.6	41 (−121;200)		133 (−269;601)	388 (−624;1445)	9.5
South Savo	−130 (−360;61)	−163 (−587;160)	−190 (−850;314)	-	−131 (−381;73)		−171 (−767;263)	−174 (−1611;880)	-
Central Finland	−29 (−266;172)	−57 (−547;315)	−90 (−900;493)	-	−33 (−304;189)		−72 (−798;434)	−222 (−2138;1051)	-
North Karelia	178 (5;354)	309 (20;679)	485 (39;1082)	2.7	193 (10;372)		428 (40;953)	1098 (162;2133)	5.7
North Savo	87 (−136;299)	171 (−199;590)	284 (−275;966)	3.5	100 (−143;329)		256 (−248;842)	721 (−543;1995)	7.2
North Ostrobothnia	40 (−216;269)	70 (−400;514)	111 (−653;826)	2.8	45 (−252;300)		97 (−560;705)	245 (−1501;1649)	5.4
Lapland	−9 (−191;141)	− 20 (−409;276)	−38 (−738;455)	-	−11 (−246;165)		−31 (−589;374)	−103 (−1663;898)	-

The results in Table 1 show heterogeneity in the projected all-age heat-related deaths between the counties. All counties show increases in heat-related excess deaths for both modelling options by 2090-99 except Lapland, Central Finland, South Savo, South Ostrobothnia, West Uusimaa, Central Uusimaa and Ostrobothnia. In those counties that exhibit an increasing trend, under the SSP5-8.5 scenario the deaths attributable to heat is higher compared to those under the SSP2-4.5 scenario. By the end of the century the heat-related mortality can be multiple times higher than in the baseline climate e.g. in South Karelia 2.7 time higher in SSP2-4.5 scenario and 5.4 times higher in the SSP5-8.5 scenario. However, the confidence intervals of the results are wide, thus the uncertainties are high with only few counties with statistically significant increase in heat-related excess deaths.

As Helsinki represents a county with high population and a low number of days with missing data (see Table SM1 in Supplementary Material) we focused our further analysis on estimating future changes in heat-related deaths for Helsinki. Our analysis indicates that estimates of heat-related deaths for the period 2010–2019 in Helsinki differ across the different modelling options due to choices in the models’ spline specifications. Using fixed knots, the excess deaths in Helsinki are 107(−164;368) and 136 (−156:449) for SSP2-4.5 and SSP5-8.5, respectively (Table 1). Respective values for the same scenarios using qAIC knots (Table SM6) are − 66 (−387;222) and − 26 (−359;301). Comparisons of projections for the end of the century (2090–2099) under the same scenarios show respective estimates of excess deaths by fixed knots of 540(−181;1647) and 1577(−85;3890) and by qAIC knots of 502 (−324;1687) and 2183 (244;5078). The wide range of confidence intervals illustrates the large uncertainty in these projections. Based on recent more detailed analyses using Helsinki mortality statistics that included additional heat events up to 2019, the fixed knots spline model was found to out-perform the qAIC knots model (Ruuhela et al. 2021; Kivimäki et al. 2023).

For cold-related mortality the results are less ambiguous than for heat. To illustrate, comparing cold AN values for Helsinki using the qAIC knots model (Supplementary Table SM7-8) cold-related mortality decreases under both scenarios and for both time periods, with confidence intervals that do not include negative values. For example, excess deaths projected for Helsinki in 2090–2099 under the two scenarios (SSP2 and SSP5) are in the qAIC model 4286 (741;7445) and 3402(275;6223) and fixed knots model 4819(1422;8014) and 4013(1070;6805).

### Accounting for population changes and adaptation

The previous projections on temperature-related mortality were based only on the increase in temperature, here we expand and elaborate the study to include impacts of population growth and adaptation scenarios on temperature-related deaths in Helsinki.

**Fig. 5:**
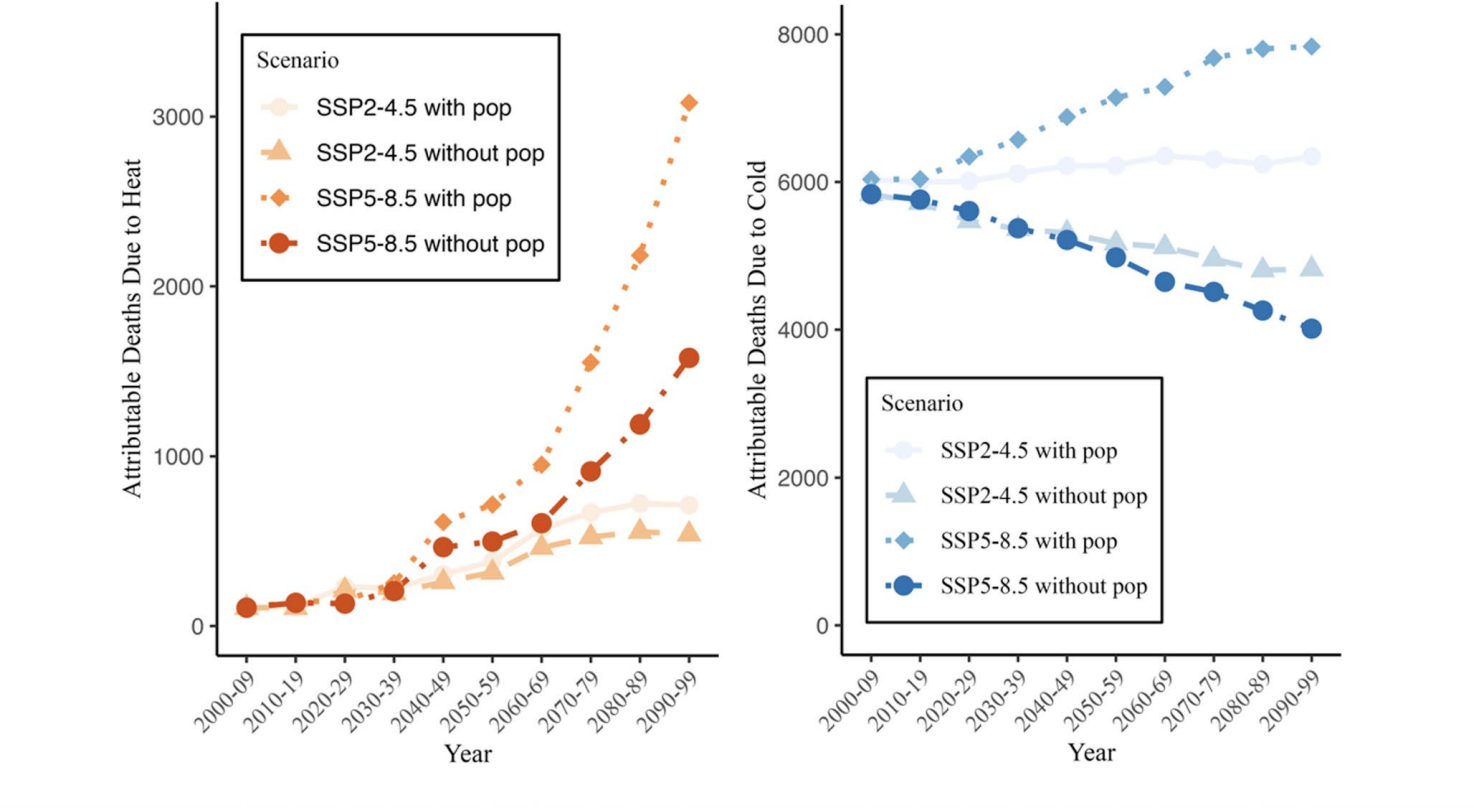
Helsinki all-cause, all-age number of deaths attributable to heat (left) and cold (right) with and without IIASA population development scenarios under SSP2-4.5 and SSP5-8.5 scenarios under fixed knots model

Figure 5 illustrates the number of deaths attributable to heat and cold in Helsinki for ten-year time intervals with and without population development under both the SSP2-4.5 and SSP5-8.5 scenarios. Including population growth in the modelling increases the total number of deaths attributable to both heat and cold as the total population increases under both future scenarios. In the absence of population growth, cold-related deaths would decrease in Helsinki as climate becomes warmer. Overall, when accounting for both climate warming and population growth, the future burden of cold-related deaths still remains higher than that of heat-related deaths in Helsinki for both SSP2-4.5 and SSP5-8.5, although heat-related deaths increase more rapidly than cold-related deaths.

**Fig. 6:**
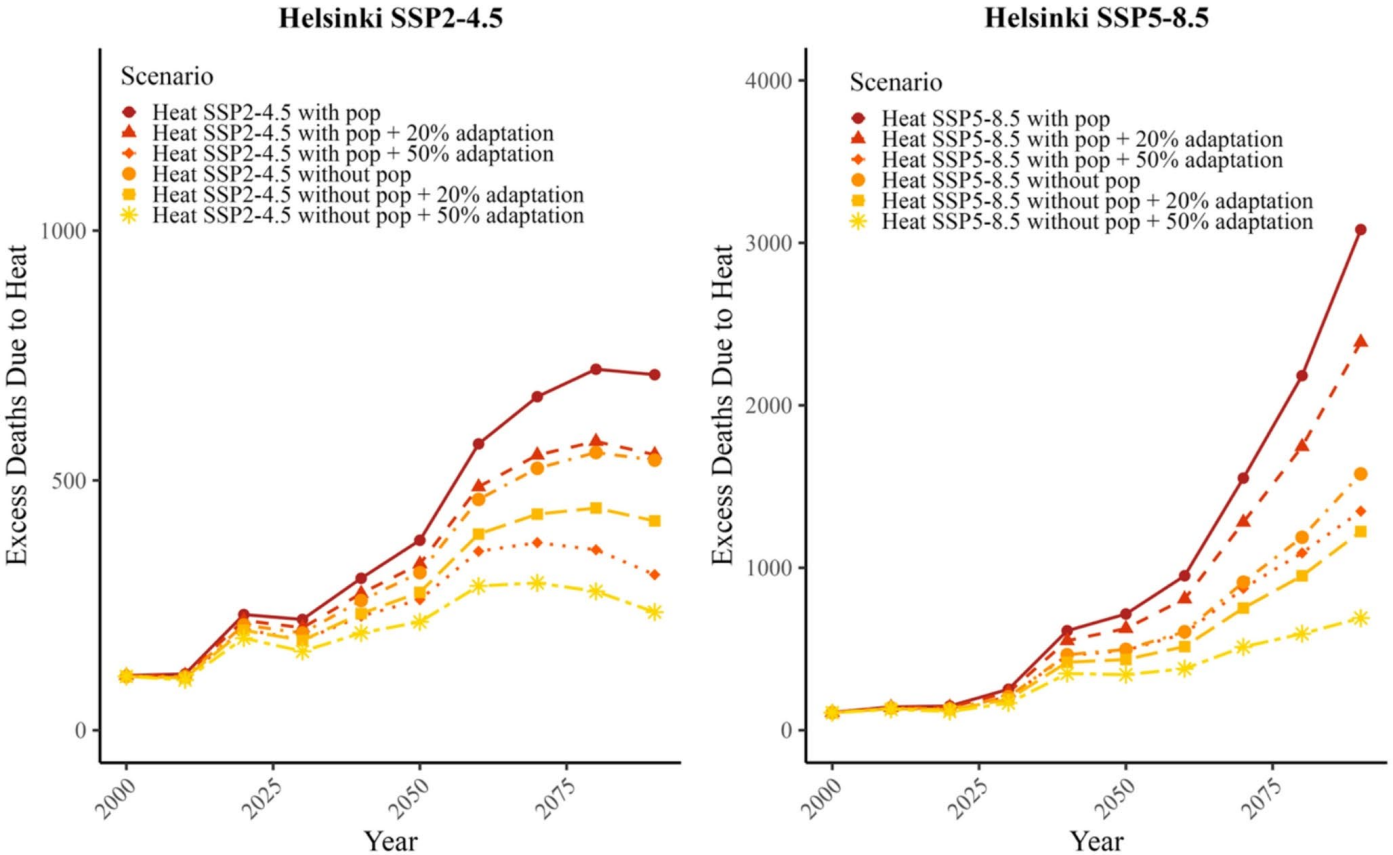
The number of deaths attributable to heat in Helsinki under fixed knots model in SSP2-4.5 (left) and SSP5-8.5 (right) climate projections considering different adaptation scenarios and population projections (pop). The reduction of the heat relative risk (RR) in the exposure-response relationship in Helsinki with adaptation scenarios of no adaptation, 20% and 50% adaptation, based on the relationship in the period 2000–2017

Figure 6 (bottom) illustrates the temperature-mortality relationship in Helsinki based on data from 2000 to 2017 with different assumptions about the level of adaptation to heat: no adaptation (0%) and 20% and 50% adaptation. Additionally, Fig. 6 illustrates the number of excess deaths attributable to heat in Helsinki in various future scenarios and with or without population development scenarios. We derived estimates until end of the century as ten-year heat-related attributable numbers of deaths (AN) under SSP2-4.5 and SSP5-8.5 climate change projections with the three adaptation scenarios. Our result projects between 270 and 3082 excess heat-related deaths by 2090–2099 depending on the combined climate change, adaptation and population scenario, compared to 107 deaths in 2010–2019. The excess number of heat-related deaths under a moderate climate change projection and 50% adaptation level in Helsinki is estimated to at least double, but is six times higher under SSP5-8.5 if no adaptation occurs. Population increase can have a large impact on estimated future heat-related deaths, as well.

## Discussion

This study examined future projections of excess heat- and cold-related mortality across different wellbeing service counties in Finland with the primary focus on heat-related impacts. Our findings indicate an increase in the heat-related deaths in 12 out of 19 counties with strongest increases towards the end of this century. Future excess cold-related deaths decreased in most of the study regions. However, population projections influence substantially the anticipated cold-related mortality, as we demonstrated for Helsinki. The results need to be interpreted with caution because of high uncertainty in the modelled relationships especially in the less-populated wellbeing counties.

Our results of projected increases in excess heat-related deaths (Table 1) support findings from other similar mortality projection studies. In China, projections of heat-related mortality in 161 Chinese cities were studied with 28 global climate models (GCMs) and the results showed that excess deaths might increase in the future from 2.3 to 5.8-fold (Yang et al. 2021). In Canada, a similar modelling approach found that most of the future temperature-related mortality will be cold-related, but heat-related deaths will increase with increasing temperatures (Hebbern et al. 2023). A study from Switzerland estimated that both heat- and cold-related deaths increase under all climate change scenarios with cold-related deaths influenced by the growth of population (de Schrijver et al. 2023b). As part of a study of European cities, Masselot et al. (2025) estimated that while future excess heat-related death rates are expected to increase in Finland, these numbers are lower than the reduction in cold-related deaths with a slight net effect of a decline in temperature-related deaths by 2095–2099 under SSP3-7.0.

Several of these studies used pooling methods and provided associations for wider areas, an approach also taken earlier in Finland by Ruuhela et al. (2018). However, the objective in this study was to analyse each county separately to assess regional variations of heat and cold risk for supporting county and municipal-scale adaptation. We note that even though models for some regions did not indicate increased risks of heat-related mortality (Supplementary Fig. SM1), the choice of age group might influence relative risks. For instance, impacts of heat on vulnerable groups in Finland have been found in an earlier study to be less well defined and unevenly distributed across different welfare areas (Astone and Vaalavuo 2022;2023).

Projections of future excess temperature-related deaths are influenced by various confounding factors including socioeconomic, built environment and public health factors that impact vulnerability to heat, rather than changes in temperatures alone. For instance, in a longitudinal study from 1972 to 2009 across 211 different cities Sera et al. 2020 found that increased air conditioning prevalence was associated with a 16.7% reduction in heat-related mortality in the United States. Urban planning is an important contributor to heat adaptation plans, affecting the environment to which people are exposed and influencing their resilience to heat. For instance, adding to urban tree coverage can benefit the health of citizens and create more sustainable and climate-resilient cities (Iungman et al. 2023). Cold-related deaths can also be connected to other confounding factors, including influenza incidence, during the winter months (Ballester et al. 2016; Arbuthnott et al. 2018). Mortality risks associated directly with cold appear at extreme low temperatures, while at milder temperatures, occurrences may be related more to indirect effects such as deaths caused by spread of infections (Arbuthnott et al. 2018).

The study has several limitations. We had a relatively short mortality time series and high number of missing death data (Table SM1) due to data protection designed to ensure anonymity in several counties. Also, the lack of adequate population projections limits the research to estimate future projections at wellbeing service county level. The mortality data available for this study included years 2000–2017 and these time series included only three longer heatwaves, in summers 2003, 2010 and 2014. Inclusion of summers with heatwave events 2018, 2019 and 2021 might change the temperature-mortality relationship especially for regions with smaller population such as South Ostrobothnia and South Savo. Thus, further research with extended time series is needed to elaborate future heat- and cold-related mortality risks.

Models developed for a number of counties failed to replicate the conventional U-shape temperature-mortality relationship for the baseline period. Consequently, this led to projections for some counties of decreasing heat-related mortality trends with future warming, which would appear to be a counter-intuitive result based on knowledge from other regions. In addition, estimates of attributable excess deaths due to heat (AN) have wide confidence intervals, reflecting high uncertainty in the results. Similar wide CIs have been reported in previous Finnish heatwave mortality studies, for instance, for age groups < 65 years(Kollanus et al. 2021). A study by Kivimäki et al. (2023) reported a statistically significant association between cardiovascular deaths in Finland and temperatures above 21 °C, though there was no significant association for non-cardiovascular deaths.

A model sensitivity analysis using alternative knot selections indicated that number of knots has a substantial influence on results for Lapland and West Uusimaa. In future studies the association could be elaborated by using longer time series, data on causes of deaths and different age groups. Furthermore, aggregating wellbeing county level data into larger areas, especially in Southern Finland, might improve modelling as well. However, our modelling suggests heterogeneous exposure–response relationships across regions and sub-national temperature-mortality modelling can provide essential local information for climate change adaptation planning.

In the regions of South Savo, Central Finland and South Ostrobothnia heat-related deaths did not increase using either of the modelling alternatives. Although Ruuhela et al. (2018) concluded that a single temperature-mortality relationship could be applied to the whole country, the meta-regression analysis in their analysis indicated that there is regional heterogeneity in the relationship, which they found could be explained by a morbidity index. The index includes factors such as the prevalence of chronic diseases that make people sensitive to heat stress. In Finland, there are regional differences in public health, and the morbidity index is lower in the western part of the country than in the east (THL, 2025).

Including population scenarios in the analysis proved to be relevant for future projections of temperature-related mortality but in Finland population projections are still not available out to 2100 for the wellbeing service counties. This underscores the need for better future projections of indicators describing vulnerability and exposure to climate change, recognizing the potential impacts of the evolving socio-economic context (Pedde et al. 2024). A narrow approach to assessing climate change risks and adaptation assessments, focusing only on future climate hazards, could lead to maladaptation and injustice (Prall et al. 2023). Following our analysis, more recent population projections were released (data available at https://data.ece.iiasa.ac.at/ssp; (KC et al. 2024) that show almost the same population growth for Finland under both SSP2 and SSP5 by 2100. These newer values have population estimates of 5.53 million for SSP5 and 5.45 million for SSP2 in 2100, but explanations for these large differences are not yet published. We decided to keep version 2 projections, as they provide a useful indication of the sensitivity of excess death estimates to a very strong increase in future population.

The method to estimate future daily deaths in Helsinki is simplistic when considering how human life expectancy and the population will change in Helsinki compared to the rest of Finland due to urbanization. Statistics Finland expects that mortality will decline until 2070 and the life-expectancy of men lengthen by almost five years and women by almost three years (OSF 2019). We use assumptions of 0%, 20% and 50% adaptation to heat to estimate what effect this could have on future heat-related deaths. The 50% level, based on past experience in Helsinki, can be considered as one plausible adaptation in the future as well, since effective adaptation measures and preparedness for heatwaves in Finnish society, including heat-health action plans in the healthcare and social welfare sector, are still only under development during the time of this study. Furthermore, limits of human physiological acclimatization and adaptation (e.g.(Vanos et al. 2023) are not likely to be exceeded during this century in Finland (Ruosteenoja and Jylhä, 2021). However, there is no definitive scenario on the future level of adaptation to heat. Furthermore, it was beyond the scope of this study to assess the impact of an aging population on future temperature-related mortality.Lipsanen et al. 2025 prepared national narrative extensions of the global shared socioeconomic pathways (SSPs) for healthcare and social wellbeing in Finland, which are designed to inform adaptation. In future research we hope to elaborate our modelling using quantitative indicators derived from interpretation of these qualitative SSP-based socioeconomic narratives.

Our modelling for Helsinki does not consider the urban heat island (UHI) effect, because neither global climate models nor interpolated FMI_ClimGrid data can describe well enough local climate effects such as UHI. The intensity of UHI in the future depends on urbanization and characteristics of the urban design. Credible scenarios for future urban sprawl are not available for Helsinki. However, Ruuhela et al. (2021) showed that for the current climate and urban structure the mortality during heatwaves in Helsinki can be about 2.5 times the mortality in the surrounding more rural areas. In future studies we aim to be able to integrate projections of urban development and of climate to improve scenarios of exposure to heat in the city.

In the study we did not assess temperature-related morbidity, but previous studies show that longer heatwaves, rather than single hot days, also affect morbidity in a high latitude climate. For example, increased respiratory admissions have been observed in Helsinki metropolitan area during prolonged heatwaves among the age group over 75 years old (Sohail et al. 2020). Causes of heat-related deaths may also shift – for instance, heat-related cardiovascular mortality is projected to increase in Finland by more than 1.5 times under climate projections assuming high-end emissions compared to those under low emissions (Kivimäki et al. 2023). For a more comprehensive understanding of health effects under non-optimal temperatures in the future, projections of morbidity related risks should also be estimated.

## Conclusion

Our study indicates that regional heat-related mortality for all-age population in Finland is expected to increase by the end of the century in the majority of wellbeing service counties, especially in the southern and eastern part of Finland. This result is valid for climate projections assuming both medium and high emissions. Cold-related mortality will decrease in most counties, but based on results from Helsinki, by including projections of population growth, the number of deaths attributable to cold may also increase. However, all results should be interpreted with caution due to limitations in availability and duration of the mortality time series, which affect statistical reliability. However, as found in most other European countries, our results confirm that strategic planning of adaptation measures to mitigate heat-related health risks is also of relevance in Finland.

## Supplementary Information

Below is the link to the electronic supplementary material.

Supplementary Material 1 (DOCX 10.1 MB) 

## Data Availability

Mortality data are not publicly available but may be accessed by submitting a request to Findata. The meteorological and climate datasets generated during and analysed during the study are available from the corresponding author on reasonable request.
