# Supplementary material for: Future temperature-related mortality in various climate change and adaptation scenarios in Finland
Source: Int J Biometeorol. 2026 Mar 2;70(3):79. doi: 10.1007/s00484-025-03105-0 (PMC12953278; doi:10.1007/s00484-025-03105-0)
Supplement: Supplementary file 1 — Supplementary Material 1 (DOCX 10.1 MB) [file 484_2025_3105_MOESM1_ESM.docx]

**Supplementary material**

# **CMIP6 Climate Models**

The five CMIP6 models selected for this study were CanESM5-r4, CMCC-CM2-SR5, GFDL-ESM4-r1, MPI-ESM1-2-HR-r1 and CNRM- CM6-1-r3. These models were selected so that the simulated future changes in average temperature and precipitation in Finland would at least be reasonably representative of the changes produced by the larger set of 28 CMIP6 models. All models have scored quite well in previous assessments of the quality of the models. From the bias-corrected climate models, we used daily average temperature values from the time period 1 January 1970 until 31 December 2099 for the forcing scenarios SSP2-4.5 and SSP5-8.5. The grid spacing from these models are 0.5°× 0.5°, which is approximately at the 60^th^ latitude 56*28 km.

# **Minimum mortality temperatures, missing data on all-age mortality and total population in the Finnish wellbeing service counties**

**Table SM1** Minimum mortality temperatures, missing data on all-age mortality and total population in the Finnish wellbeing service counties. Counties with percentage missing data above 50% were excluded from some analyses.

| **Wellbeing service county** | **Minimum mortality temperature (MMT)** | **Missing data on all-age mortality**.  **(2000-2017) and (%)** | **Total Population**  **(in 2017)** |
| --- | --- | --- | --- |
| Central Finland | 15 | 196 (2.98%) | 276570 |
| Central Ostrobothnia | 15 | **4992 (75.9%)** | 69440 |
| Central Uusimaa | 16 | 2396 (36.4%) | 197458 |
| East Uusimaa | 16 | **3916 (59.6%)** | 98082 |
| Helsinki | \| 16 \| \| --- \| | 2 (0.03%) | 648268 |
| Kainuu | 14 | **3471 (52.8%)** | 74952 |
| Kanta-Häme | 15 | 925 (14.1%) | 174542 |
| Kymenlaakso | 15 | 481 (7.32%) | 170850 |
| Lapland | 14 | 762 (11.6%) | 181313 |
| North Karelia | 15 | 661 (10.1%) | 168520 |
| North Ostrobothnia | 14 | 66 (1.00%) | 415154 |
| North Savo | 15 | 112 (1.7%) | 254508 |
| Ostrobothnia | 15 | 1169 (17.8%) | 178030 |
| Päijät-Häme | 15 | 464 (7.06%) | 210378 |
| Pirkanmaa | 15 | 2 (0.03%) | 519138 |
| Satakunta | 15 | 208 (3.16%) | 223181 |
| South Karelia | 15 | 1345 (20.5%) | 131497 |
| South Ostrobothnia | 15 | 460 (7.00%) | 197755 |
| South Savo | 15 | 799 (12.2%) | 140796 |
| Southwest Finland | 16 | 1 (0.02%) | 482586 |
| Vantaa and Kerava | 16 | 1963 (29.9%) | 260203 |
| West  Uusimaa | 16 | 163 (2.48%) | 463542 |
| Åland | 16 | 6342 (96.5%) | 29720 |

# **Root Mean Square Error (RMSE) and relative RMSE for fixed knot model and qAIC knot alternative model under lag 21**

**Table SM2** Fixed knots model and qAIC knots model RMSE and relative RMSE model for deaths estimates. The relative RMSE is normalized by mean of all deaths.

| **Counties** | **RMSE**  **fixed kntos**  **model** | **Relative**  **RMSE**  **Fixed knots**  **model*** | **%** | **RMSE**  **qAIC model** | **Relative**  **RMSE**  **qAIC**  **model*** | **%** |
| --- | --- | --- | --- | --- | --- | --- |
| Central Finland | 2,756 | 0,400 | 40,0 | 2,752 | 0,399 | 39,9 |
| Central Ostrobothnia | 1,091 | 0,340 | 34,0 | 1,087 | 0,339 | 33,9 |
| Central Uusimaa | 1,460 | 0,371 | 37,1 | 1,458 | 0,371 | 37,1 |
| East Uusimaa | 1,195 | 0,363 | 36,3 | 1,192 | 0,362 | 36,2 |
| Helsinki | 3,836 | 0,289 | 28,9 | 3,851 | 0,290 | 29,0 |
| Kainuu | 1,325 | 0,374 | 37,4 | 1,324 | 0,374 | 37,4 |
| Kanta-Häme | 2,157 | 0,433 | 43,3 | 2,155 | 0,433 | 43,3 |
| Kymenlaakso | 2,322 | 0,417 | 41,7 | 2,322 | 0,417 | 41,7 |
| Lapland | 2,227 | 0,436 | 43,6 | 2,228 | 0,436 | 43,6 |
| North Karelia | 2,222 | 0,424 | 42,4 | 2,224 | 0,424 | 42,4 |
| North Ostrobothnia | 3,046 | 0,383 | 38,3 | 3,045 | 0,383 | 38,3 |
| North Savo | 2,850 | 0,391 | 39,1 | 2,850 | 0,391 | 39,1 |
| Ostrobothnia | 1,853 | 0,388 | 38,8 | 1,853 | 0,388 | 38,8 |
| Päijät-Häme | 2,424 | 0,417 | 41,7 | 2,423 | 0,417 | 41,7 |
| Pirkanmaa | 3,655 | 0,306 | 30,6 | 3,652 | 0,306 | 30,6 |
| Satakunta | 2,696 | 0,403 | 40,3 | 2,696 | 0,403 | 40,3 |
| South Karelia | 1,845 | 0,406 | 40,6 | 1,851 | 0,407 | 40,7 |
| South Ostrobothnia | 2,468 | 0,434 | 43,4 | 2,467 | 0,434 | 43,4 |
| South Savo | 2,174 | 0,422 | 42,2 | 2,174 | 0,421 | 42,1 |
| Southwest Finland | 3,586 | 0,300 | 30,0 | 3,581 | 0,299 | 29,9 |
| Vantaa and Kerava | 1,698 | 0,411 | 41,1 | 1,697 | 0,410 | 41,0 |
| West Uusimaa | 2,829 | 0,405 | 40,5 | 2,829 | 0,405 | 40,5 |

# **Temperature-mortality relationship and temperature histogram in Finnish wellbeing service counties with fixed knots and qAIC based knots for the lag 21 (lag 14, and 7 available on request)**

## **Temperature-mortality relationships for fixed knots model**

**
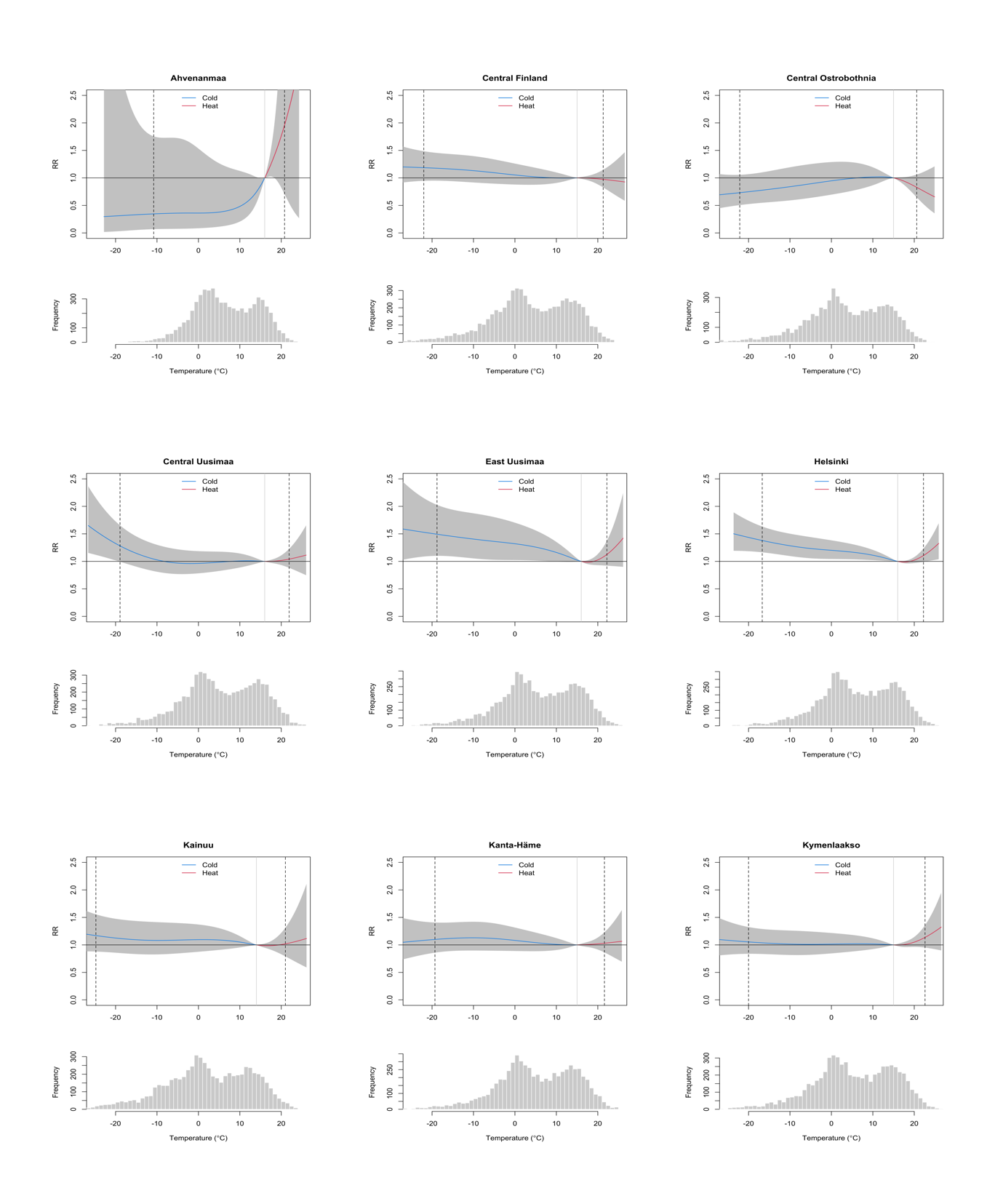
**

**
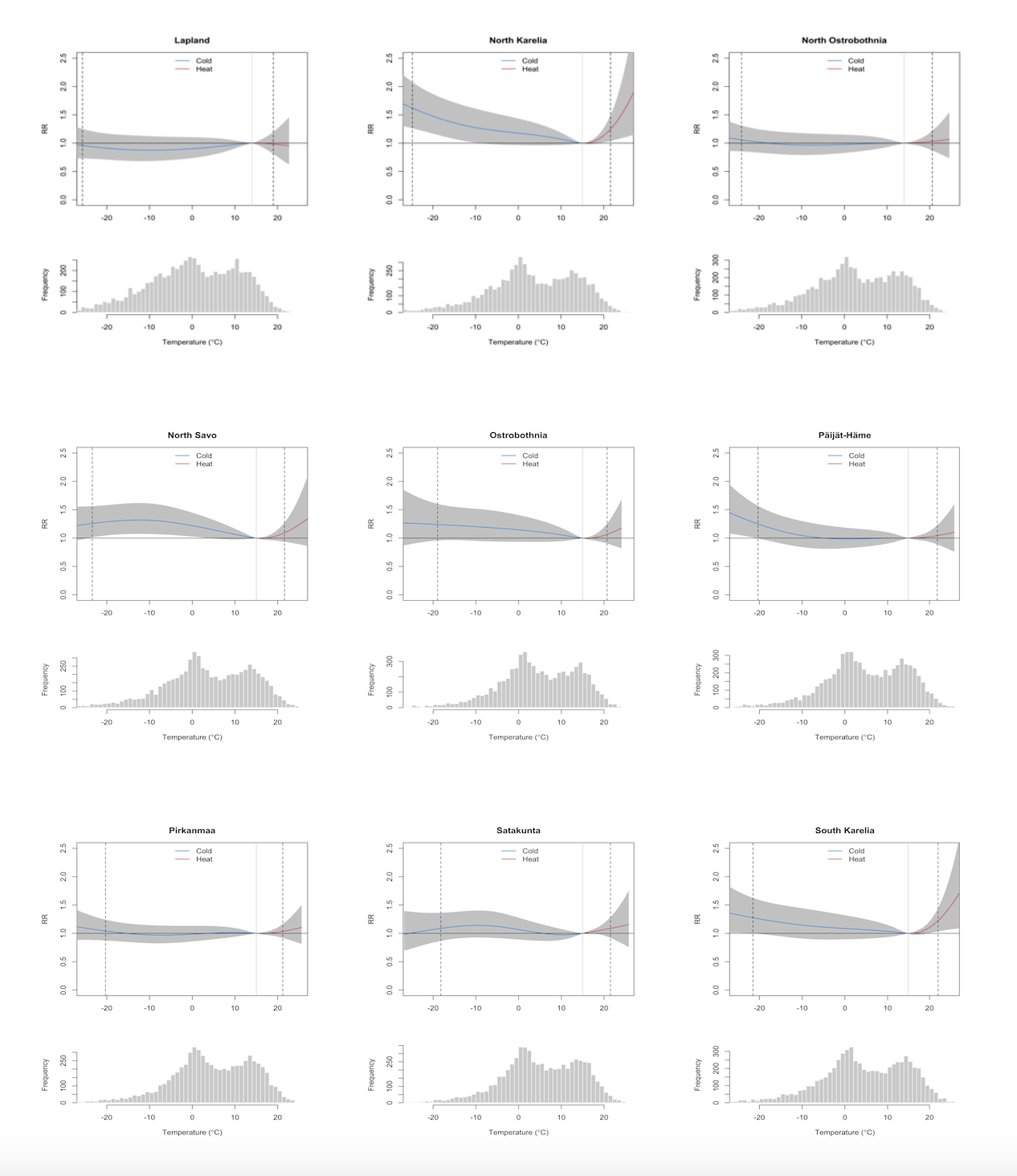
**

**
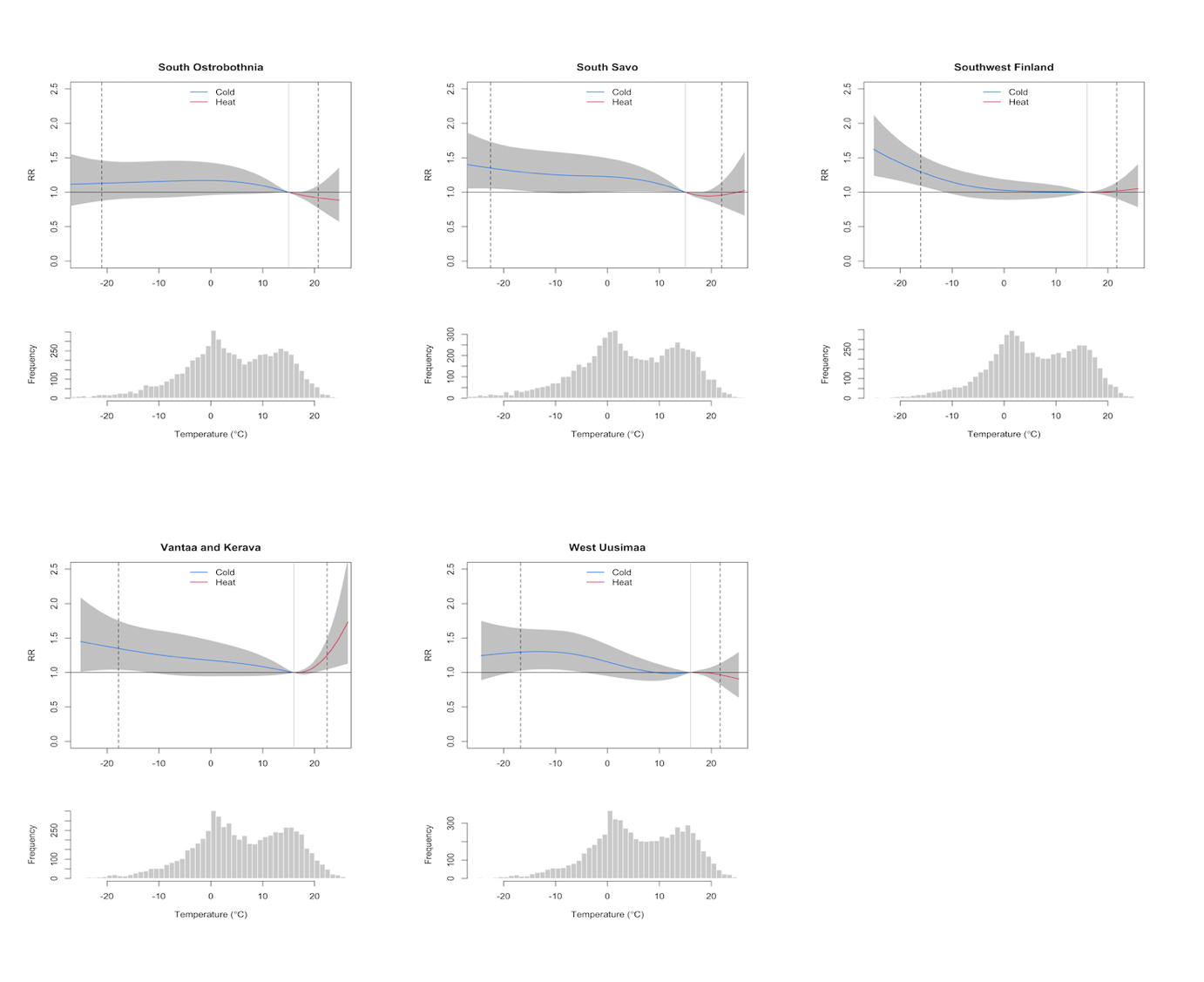
**

## **Fig. SM1** Overall cumulative relative risks (RR) of temperature-related mortality and daily mean temperature distributions in Finnish wellbeing service counties in 2000-2017. The upper graphs the grey area illustrates the 95%-CI (confidence interval) of the modelled relationship*.*The dashed vertical lines present 1^st^ and 99^th^ percentiles and MMT is presented as solid vertical line. The lower graphs present the frequency distribution of the daily mean temperature. The results are presented for fixed knots model under the lag 21.

## **Temperature-mortality realtionships for qAIC based knot model**

**
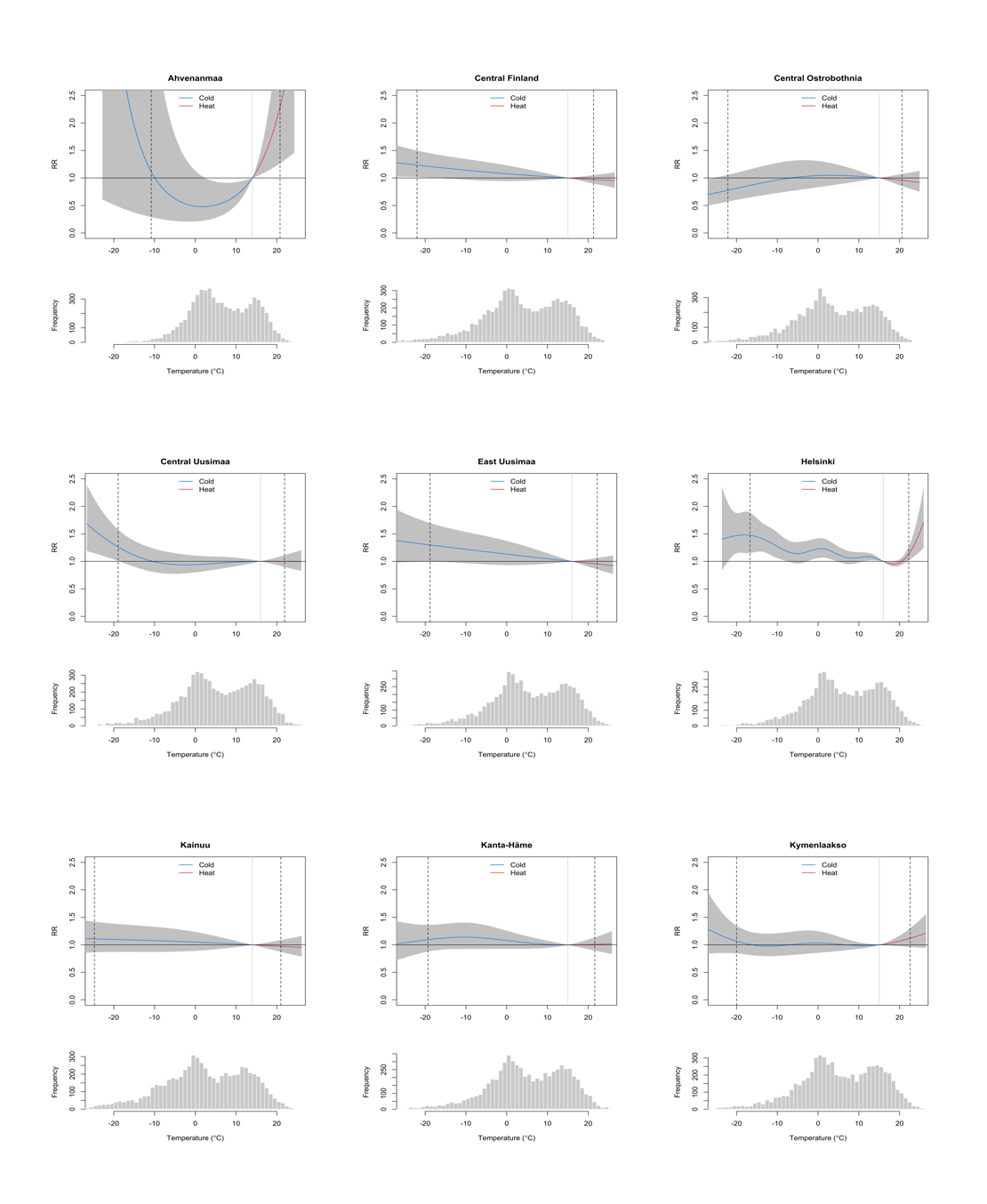
**

**
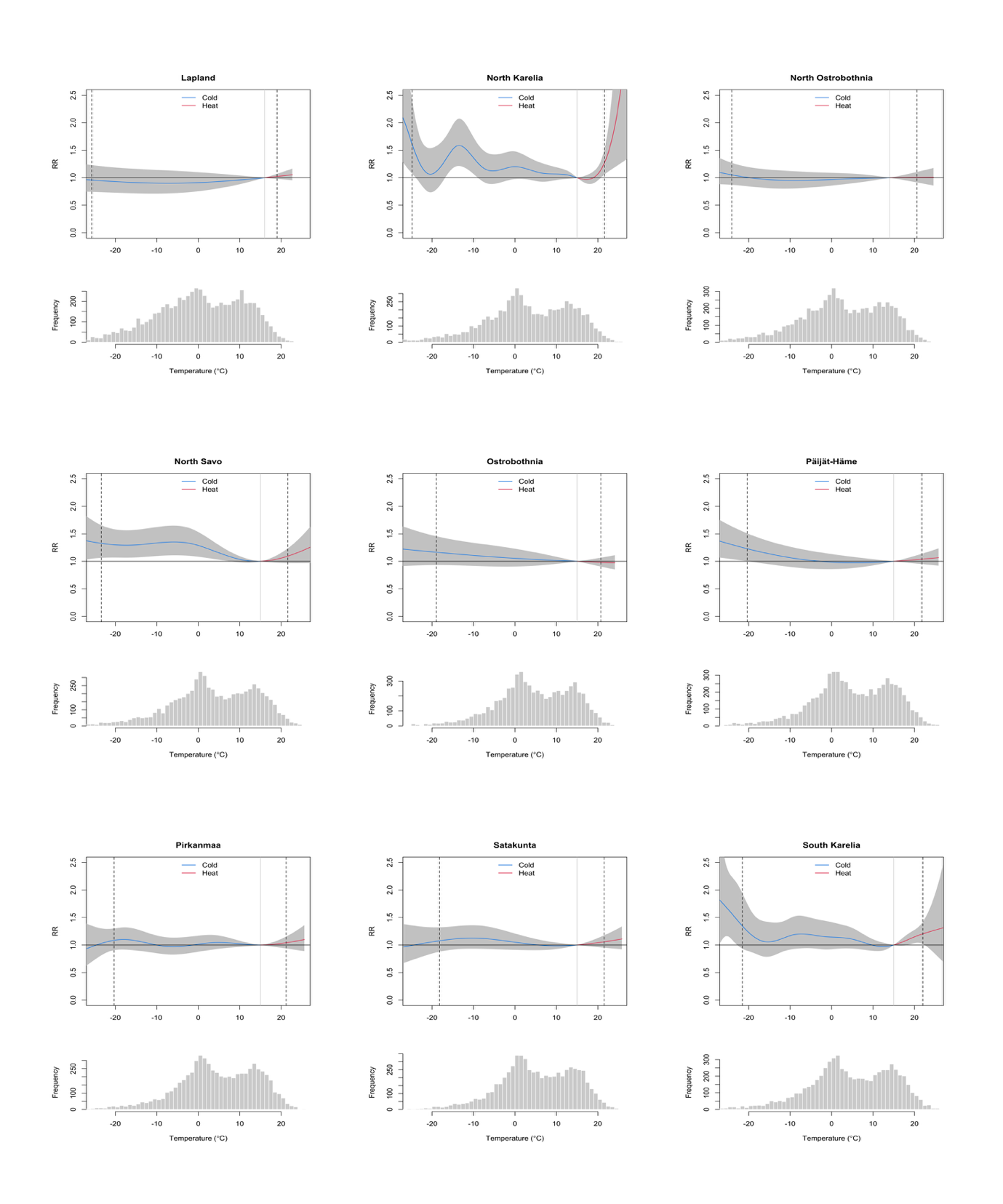
**

**
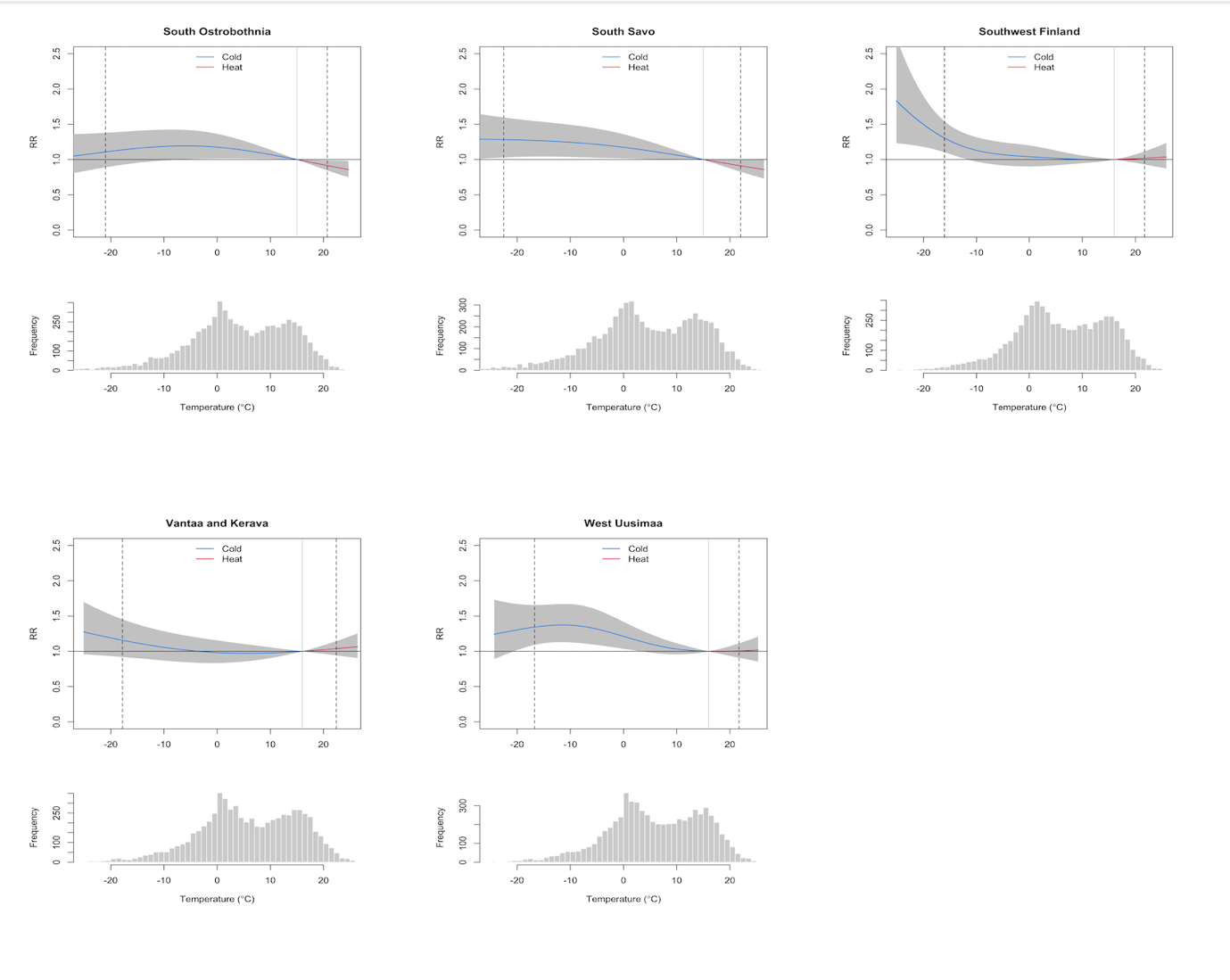
**

**Fig. SM2** Overall cumulative relative risks (RR) of temperature-related mortality and daily mean temperature distributions in Finnish wellbeing service counties in 2000-2017. The upper graphs the grey area illustrates the 95%-CI (confidence interval) of the modelled relationship*.*The dashed vertical lines present 1^st^ and 99^th^ percentiles and MMT is presented as solid vertical line. The lower graphs present the frequency distribution of the daily mean temperature. The results are presented for the QAIC based knots under the lag 21.

|  |  |  |  |
| --- | --- | --- | --- |
|  |  |  |  |
|  |  |  |  |
|  | |  |  |

# **Quasi-Akaike Information Criterion (qAIC) for the best knot selection for the DLNM modelling for 21, 14 and 7 days lag**

**Table SM3 Quasi-Akaike Information Criterion (**qAIC) for the best knot selection for the DLNM modelling

| **Wellbeing service county** | **Best knot for the variable (temperature)** | **Best knot for the lag** |
| --- | --- | --- |
| Central Finland | 1 | 1 |
| Central Ostrobothnia | 1 | 1 |
| Central Uusimaa | 2 | 2 |
| East Uusimaa | 1 | 1 |
| Helsinki  Ahvenanmaa/Åland | 7  1 | 2  4 |
| Kainuu | 1 | 2 |
| Kanta-Häme | 2 | 1 |
| Kymenlaakso | 3 | 2 |
| Lapland | 1 | 2 |
| North Karelia | 8 | 1 |
| North Ostrobothnia | 2 | 1 |
| North Savo | 3 | 2 |
| Ostrobothnia | 1 | 2 |
| Päijät-Häme | 2 | 1 |
| Pirkanmaa | 4 | 1 |
| Satakunta | 2 | 5 |
| South Karelia | 7 | 2 |
| South Ostrobothnia | 1 | 2 |
| South Savo | 1 | 1 |
| Southwest Finland | 3 | 1 |
| Vantaa_Kerava | 1 | 1 |
| West Uusimaa | 2 | 1 |

**Table SM4 Quasi-Akaike Information Criterion (**qAIC) for the best knot selection for the DLNM modelling for 14 days lag

| **Wellbeing service county** | **Best knot for the variable (temperature)** | **Best knot for the lag** |
| --- | --- | --- |
| Central Finland | 1 | 1 |
| Central Ostrobothnia | 1 | 1 |
| Central Uusimaa | 3 | 1 |
| East Uusimaa | 1 | 1 |
| Helsinki | 7 | 1 |
| Kainuu | 1 | 1 |
| Kanta-Häme | 2 | 2 |
| Kymenlaakso | 3 | 3 |
| Lapland | 1 | 1 |
| North Karelia | 8 | 1 |
| North Ostrobothnia | 1 | 1 |
| North Savo | 3 | 1 |
| Ostrobothnia | 1 | 1 |
| Päijät-Häme | 1 | 5 |
| Pirkanmaa | 4 | 1 |
| Satakunta | 2 | 1 |
| South Karelia | 7 | 2 |
| South Ostrobothnia | 1 | 1 |
| South Savo | 1 | 1 |
| Southwest Finland | 8 | 3 |
| Vantaa_Kerava | 1 | 1 |
| West Uusimaa | 2 | 1 |

**Table SM5 Quasi-Akaike Information Criterion (**qAIC) for the best knot selection for the DLNM modelling for 7 days lag

| **Wellbeing service county** | **Best knot for the variable (temperature)** | **Best knot for the lag** |
| --- | --- | --- |
| Central Finland | 5 | 1 |
| Central Ostrobothnia | 1 | 1 |
| Central Uusimaa | 1 | 1 |
| East Uusimaa | 1 | 4 |
| Helsinki | 7 | 1 |
| Kainuu | 1 | 1 |
| Kanta-Häme | 2 | 1 |
| Kymenlaakso | 3 | 2 |
| Lapland | 1 | 2 |
| North Karelia | 4 | 1 |
| North Ostrobothnia | 1 | 1 |
| North Savo | 3 | 1 |
| Ostrobothnia | 1 | 1 |
| Päijät-Häme | 1 | 1 |
| Pirkanmaa | 4 | 1 |
| Satakunta | 2 | 3 |
| South Karelia | 3 | 1 |
| South Ostrobothnia | 5 | 1 |
| South Savo | 1 | 2 |
| Southwest Finland | 3 | 1 |
| Vantaa_Kerava | 1 | 2 |
| West Uusimaa | 2 | 1 |

# **All-age excess heat- and cold related attributable number of deaths (AN)**

**Table SM6** All-age excess heat related attributable number of deaths (AN) for wellbeing service counties in Finland with 95% confidence interval (CI) with results based on **best qAIC** knot values under the lag 21. The results include model projections for 2010-2019, 2050-2059 and 2090-2099 under both SSP2-4.5 and SSP5-8.5.

|  |  | **AN Heat under SSP2-4.5** |  |  | **AN heat under SSP5-8.5** |  |
| --- | --- | --- | --- | --- | --- | --- |
| **Wellbeing service**  **county** | **2010-2019 SSP2-4.5** | **2050-2059 SSP2-4.5** | **2090-2099 SSP2-4.5.5** | **2010-2019 SSP5-8.5** | **2050-2059 SSP5-8.5** | **2090-2099 SSP5-8.5** |
| Central Finland | -48  (-187;84) | -76  (-323;166) | -109  (-484;200) | -52  (-204;90) | -96  (-428;171) | -205  (-898;361) |
| **Central Uusimaa** | 2  ( -100;95) | 2  (-183;155) | 2  (-275;254) | 2  (-109;107) | 2  (-243;221) | -1  (-558;484) |
| **Helsinki** | -66  (-387; 222) | 200  (-338;897) | 502  (-324;1687) | -26  (-359;301) | 483  (-225;1485) | 2183  (244;5078) |
| **Kanta-Häme** | 8  (-154;156) | 15  (-256;266) | 23  (-362;389) | 9  (-162;171) | 21  (-329;342) | 53  (-685;723) |
| **Kymenlaakso** | 153  (-67; 357) | 240  (-94;614) | 133  (-196; 501) | 159  (-71;380) | 299  (-114;790) | 628  (-225;1567) |
| **Lapland** | 34  (-30;107) | 56  (-53;197) | 90  (-84;323) | 38  (-34;120) | 76  (-70;266) | 181  (-167;582) |
| **North Karelia** | 86  (-120;277) | 228  (-100;590) | 435  (-64;1082) | 108  (-101;304) | 382  (-30;898) | 1313  (288;2491) |
| **North Ostrobothnia** | 11  (-176;187) | 15  (-295;307) | 21  (-430;450) | 11  ( -102;300) | 18  (-382;391) | 35  (-823;802) |
| **North Savo** | 130  (-85;335) | 215  (-116;592) | 326  (-152;915) | 142  (-87;362) | 292  (-137;805) | 692  (-235;1698) |
| Ostrobothnia | -20  (-116;73) | -34  (-225;133) | -50  (-348;200) | -22  (-133;81) | -43  (-293;170) | -92  (-627;362) |
| **Päijät-Häme** | 62  (-86;213) | 97  (-133;350) | 133  (-186;501) | 85  (-89;223) | 121  (-167;444) | 250  (-332;875) |
| **Pirkanmaa** | 92  (-200;383) | 172  (-331;731) | 259  (-469;1101) | 101  (-210;421) | 235  (-427;997) | 579  (-919;2203) |
| **Päijät-Häme** | 82  (-86;253) | 141  (-137;464) | 201  (-187;668) | 89  (-89;275) | 176  (-167;610) | 392  (-363;1227) |
| **South Karelia** | 253  (50;452) | 371  (68;710) | 493  (55;1017) | 260  (50;458) | 444  (52;914) | 825  (-351;1813) |
| South Ostrobothnia | -114  (-243;-8) | -195  (-465;-15) | -287  (-716;-22) | -124  (-264;-10) | -253  (-640;-20) | -566  (-1354;-41) |
| South Savo | -140  (-305;4) | -215  (-508;6) | -297  (-746;9) | -147  (-319;4) | -268  (-678;8) | -548  (-1344;18) |
| **Southwest Finland** | 43  (-196;278) | 79  (-365;536) | 116  (-547;831) | 47  (-217;303) | 103  (-474;699) | 245  (-1090;1584) |
| **Vantaa and Kerava** | 35  (-60; 134) | 59  (-101;236) | 82  (-147; 350) | 38  (-64;147) | 74  (-127;305) | 162  (-282;634) |
| **West**  **Uusimaa** | 2  (-161; 154) | 8  (-293;297) | 16  (-435;450) | 3  (-176;175) | 14  (-379;389) | 54  (-840;884) |

**Table SM7** All-age excess cold related attributable number of deaths (AN) for wellbeing service counties in Finland with 95% confidence interval (CI) with results based on **best qAIC knot values** under the lag 21. The results include model projections for 2010-2019, 2050-2059 and 2090-2099 under both SSP2-4.5 and SSP5-8.5.

| **Wellbeing service**  **county** | **2010-2019 SSP2-4.5** | | | **2050-2059 SSP2-4.5** | | | **2090-2099**  **SSP2-4.5** | | | **2010-2019**  **SSP5-8.5** | | | **2050-2059**  **SSP5-8.5** | | | **2090-2099**  **SSP5-8.5** | | |  |  |
| --- | --- | --- | --- | --- | --- | --- | --- | --- | --- | --- | --- | --- | --- | --- | --- | --- | --- | --- | --- | --- |
| **Central Finland** | | 1434  (-821;3220) | | | 1249  (-811;3082) | | | 1151  (-774;2876) | | | 1453  (-793;3345) | | | 1187  (-792;2982) | | | 876  (-733;2331) | | |  |
| **Central Uusimaa** | | | -341  ( -1945;1113) | | | -370  (-1806;956) | | | -359  (-1702;887) | | | -307  (-1907;1144) | | | -359  (-1740;922) | | | -314  (-1419;707) | | |
| **Helsinki** | | | 5186  (1063; 8666) | | | 4620  (818;7977) | | | 4286  (741;7445) | | | 5214  (1132; 8679) | | | 4415  (690;7669) | | | 3402  (275; 6223) | | |
| **Kanta-Häme** | | 839  (-866;2393) | | | 727  (-826;2146) | | | 668  (-761;2019) | | | 838  (-853;2397) | | | 686  (-803;2059) | | | 481  (-681;1618) | | |  |
| **Kymenlaakso** | | | 154  (-2186; 2212) | | | 139  (-1978;2034) | | | 126  (-1865;1885) | | | 159  (-72;380) | | | 132  (-1910;1949) | | | 96  (-1501;1557) | | |
| **Lapland** | | -1137  (-4075;1247) | | | -1089  (-3861:1142) | | | -1029  (-3613;1056) | | | -1126  (-5051;1241) | | | -1054  (-3716;1092) | | | -911  (-3090;875) | | |  |
| **North**  **Karelia** | | 2207  (-307;4179) | | | 1985  (-296;3851) | | | 1834  (-332;3626) | | | 2227  (-216;4190) | | | 1882  (-336;3718) | | | 1467  (-387;3050) | | |  |
| **North Ostrobothnia** | | -687  (-3488;1919) | | | -663  (-3260;1753) | | | -624  (-3024;1636) | | | -666  ( -3460;1941) | | | -629  (-3121;1689) | | | -510  (-2502;1365) | | |  |
| **North**  **Savo** | | 3726  (1301;5931) | | | 3402  (1136;5489) | | | 3168  (1043;5123) | | | 3694  (1283;5885) | | | 3255  (1077;5302) | | | 2560  (731;4330) | | |  |
| **Ostrobothnia** | | 679  (-1107;2187) | | | 580  (-1066;1973) | | | 527  (-999;1805) | | | 689  (-1097;2168) | | | 547  (-1044;1900) | | | 395  (-860;1473) | | |  |
| **Päijät-Häme** | | 42  (-1990;1790) | | | -79  (-1953;1555) | | | -100  (-1844;1443) | | | 78  (-1951;1796) | | | -95  (-1889;1484) | | | -199  (-1656;1065) | | |  |
| **Pirkanmaa** | | 515  (-3558;4130) | | | 543  (-3130;3941) | | | 513  (-2914;3670) | | | 533  (-3540;4155) | | | 549  (-2986;3813) | | | 594  (-2257;3292s) | | |  |
| **Satakunta** | | 672  (-1479;2638) | | | 520  (-1414; 2316) | | | 459  (-1335;2143) | | | 677  (-1468;2664) | | | 472  (-1391;2210) | | | 234  (-1226;1648) | | |  |
| **South**  **Karelia** | | 1201  (-867;2896) | | | 1075  (-831;2692) | | | 993  (-782;2494) | | | 1198  (-871;2879) | | | 1032  (-819;2495) | | | 783  (-689;2069) | | |  |
| **South Ostrobothnia** | | 2071  (123;3737) | | | 1938  (128;3496) | | | 1808  (118;3256) | | | 2056  (107;3698) | | | 1865  (134;3389) | | | 1536  (126;2795) | | |  |
| **South Savo** | | 1945  (246;3413) | | | 1752  (194;3134) | | | 1635  (168;2924) | | | 1952  (253;3378) | | | 1682  (180;3041) | | | 1316  (106;2425) | | |  |
| **Southwest Finland** | | 1303  (-2260;4629) | | | 971  (-2354;4042) | | | 856  (-2224;3745) | | | 1387  (-2198;4688) | | | 911  (-2778;3894) | | | 532  (-2039;2972) | | |  |
| **Vantaa and Kerava** | -93  (-1777; 1312) | | | -156  (-1700;1224) | | | -161  (-1606;1077) | | | -70  (-1753;1327) | | | -161  (-1656;1187) | | | -199  (-1399;818) | | |  |  |
| **West**  **Uusimaa** | 2671  (508; 4681) | | | 2286  (280;4367) | | | 2096  (218;3926) | | | 2684  (525;4702) | | | 2163  (209;4030) | | | 1520  (-76;3084) | | |  |  |

**Table SM8** All-age excess cold related attributable number of deaths (AN) for wellbeing service counties in Finland with 95% confidence interval (CI) with results based on **fixed knots** values under the lag 21. The results include model projections for 2010-2019, 2050-2059 and 2090-2099 under both SSP2-4.5 and SSP5-8.5.

| **Wellbeing service**  **county** | **2010-2019 SSP2-4.5** | | | **2050-2059 SSP2-4.5** | | | **2090-2099**  **SSP2-4.5** | | | **2010-2019**  **SSP5-8.5** | | | **2050-2059**  **SSP5-8.5** | | | **2090-2099**  **SSP5-8.5** | | |  |  |
| --- | --- | --- | --- | --- | --- | --- | --- | --- | --- | --- | --- | --- | --- | --- | --- | --- | --- | --- | --- | --- |
| **Central Finland** | | 1039  (-2232;3653) | | | 862  (-2159;3335) | | | 782  (-2044;3113) | | | 1055  (-2178;3646) | | | 805  (-2121;3235) | | | 516  (-1941;2576) | | |  |
| **Central Uusimaa** | | | -72  ( -2183;1712) | | | -118  (-2053;1537) | | | -121  (-1954;1439) | | | -38  (-2147;1735) | | | -115  (-1990;1496) | | | -109  (-1653;1228) | | |
| **Helsinki** | | | 5720  (1856;9254) | | | 5167  (1575;8527) | | | 4819  (1422;8014) | | | 5762  (1970; 9276) | | | 4978  (1502;4242) | | | 4013  (1070;6805) | | |
| **Kanta-Häme** | | 841  (-1580;2754) | | | 734  (-1508;2532) | | | 676  (-1413;2374) | | | 840  (-1565;2677) | | | 695  (-1467;2425) | | | 498  (-1257;1951) | | |  |
| **Kymenlaakso** | | | 276  (-2656; 2580) | | | 252  (-2463;2397) | | | 238  (-2310;2272) | | | 279  (-2644;2579) | | | 247  (-2376;2316) | | | 219  (-1908;1936) | | |
| **Lapland** | | -1571  (-5368;1333) | | | -1492  (-5077:1207) | | | -1401  (-4718;1151) | | | -1556  (-5348;1316) | | | -1437  (-4900;1187) | | | -1211  (-4062;1003) | | |  |
| **North**  **Karelia** | | 2315  (-187;4275) | | | 2048  (-287;3922) | | | 1909  (-308;3669) | | | 2338  (-154;1289) | | | 1968  (-296;3799) | | | 1520  (-378;3110) | | |  |
| **North Ostrobothnia** | | -434  (-4400;2741) | | | -431  (-4157;2549) | | | -408  (-3861;2383) | | | -415  ( -4382;2745) | | | -408  (-3998;2462) | | | -337  (-3301;2059) | | |  |
| **North**  **Savo** | | 3395  (450;5773) | | | 3095  (345;5357) | | | 2890  (303;5014) | | | 3384  (451;5721) | | | 2968  (305;5187) | | | 2346  (102;4286) | | |  |
| **Ostrobothnia** | | 1528  (-742;3474) | | | 1375  (-730;3206) | | | 1269  (-691;2976) | | | 1533  (-723;3460) | | | 1341  (-712;3195) | | | 1030  (-670;2514) | | |  |
| **Päijät-Häme** | | 85  (-2766;2337) | | | -13  (-2665;2092) | | | -31  (-2520;1972) | | | 124  (-2714;2371) | | | -21  (-2593;2034) | | | -87  (-2162;1622) | | |  |
| **Pirkanmaa** | | -217  (-4720;3499) | | | -174  (-4350;3286) | | | -154  (-4045;3098) | | | -132  (-4550;3593) | | | 36  (-4174;3522) | | | 502  (-3823;1448) | | |  |
| **Satakunta** | | 781  (-2126;3287) | | | 607  (-2066;2966) | | | 534  (-1960;2746) | | | 782  (-2105;3273) | | | 551  (-2040;2842) | | | 616  (-632;1931) | | |  |
| **South**  **Karelia** | | 989  (-1165;2713) | | | 855  (-1137;2487) | | | 791  (-1104;2304) | | | 1003  (-1146;2719) | | | 818  (-1120;2390) | | | 783  (-689;2069) | | |  |
| **South Ostrobothnia** | | 2086  (-728;4273) | | | 1975  (-607;4060) | | | 1852  (-569;3795) | | | 2075  (-721;4256) | | | 1913  (-576;3943) | | | 1626  (-434;3324) | | |  |
| **South Savo** | | 2440  (109;4241) | | | 2248  (92;3974) | | | 2113  (89;3751) | | | 2445  (121;4265) | | | 2176  (87;3866) | | | 1802  (77;3220) | | |  |
| **Southwest Finland** | | 1237  (-2894;4946) | | | 902  (-2935;4413) | | | 799  (-2799;4116) | | | 1326  (-2820;5011) | | | 848  (-2871;4255) | | | 494  (-2622;3348) | | |  |
| **Vantaa and Kerava** | 1544  (-449;3199) | | | 1378  (-472;2964) | | | 1279  (-445;2764) | | | 1553  (-403;3207) | | | 1323  (-456;2845) | | | 1043  (-416;2339) | | |  |  |
| **West**  **Uusimaa** | 1885  (-1290;4473) | | | 1578  (-1453;3981) | | | 1390  (-1431;3699) | | | 1903  (-1278;4469) | | | 1436  (-1467;3837) | | | 891  (-1557;2940) | | |  |  |

# **Wellbeing service counties of Finland**


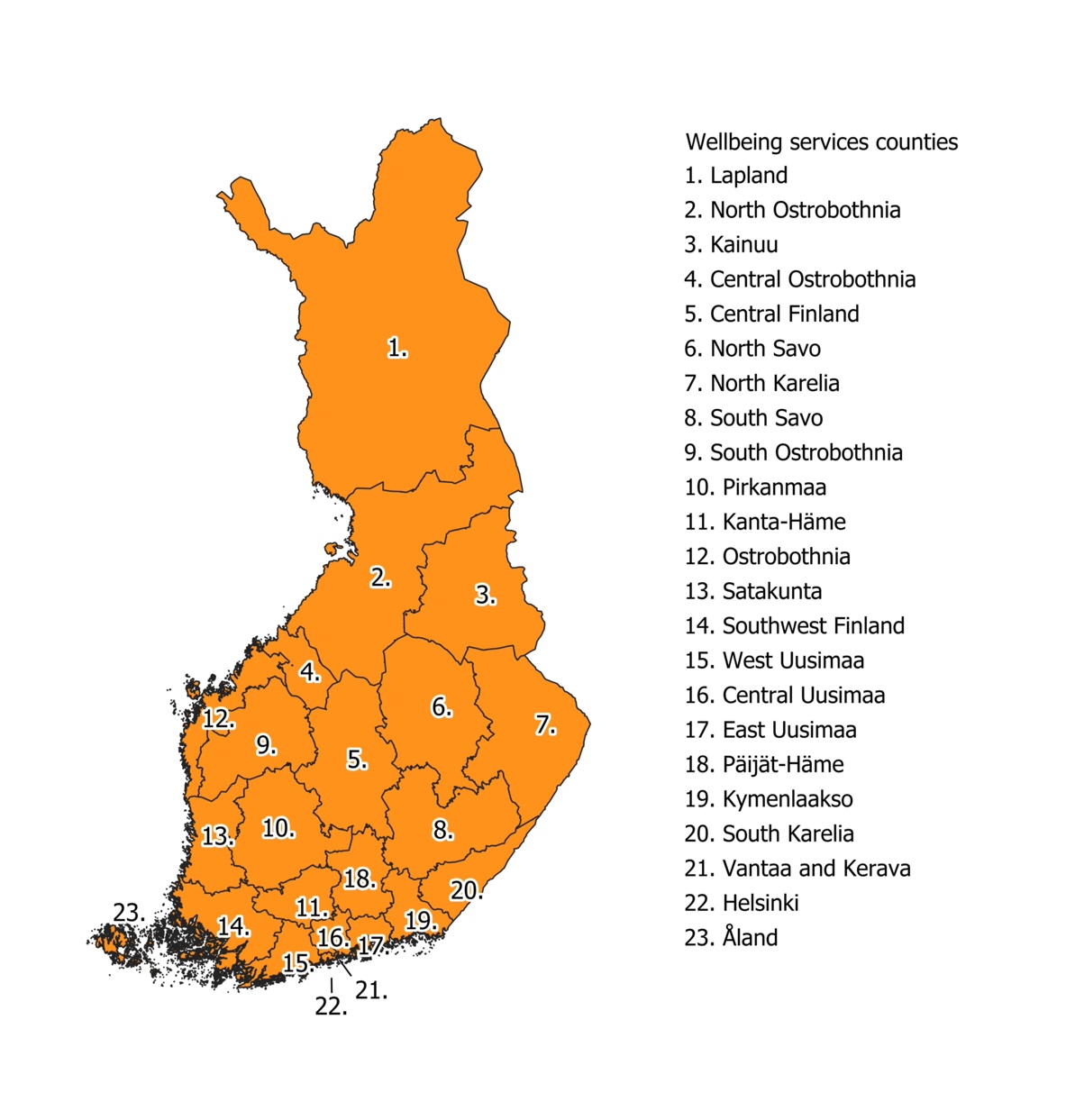


**Fig. SM3** Wellbeing service counties of Finland
